# Existence, uniqueness and regularity of the projection onto differentiable manifolds

**DOI:** 10.1007/s10455-021-09788-z

**Published:** 2021-07-01

**Authors:** Gunther Leobacher, Alexander Steinicke

**Affiliations:** 1 Institute of Mathematics and Scientific Computing, University of Graz, Heinrichstraße 36, 8010 Graz, Austria; 2 Institute of Applied Mathematics, Montanuniversitaet Leoben, Peter-Tunner-Straße 25/I, 8700 Leoben, Austria

**Keywords:** Nonlinear orthogonal projection, Medial axis, Sets of positive reach, Tubular neighborhood, 53A07, 57N40

## Abstract

We investigate the maximal open domain ${\mathscr {E}}(M)$ on which the orthogonal projection map *p* onto a subset $M\subseteq {{\mathbb {R}}}^d$ can be defined and study essential properties of *p*. We prove that if *M* is a $C^1$ submanifold of ${{\mathbb {R}}}^d$ satisfying a Lipschitz condition on the tangent spaces, then ${\mathscr {E}}(M)$ can be described by a lower semi-continuous function, named *frontier function*. We show that this frontier function is continuous if *M* is $C^2$ or if the topological skeleton of $M^c$ is closed and we provide an example showing that the frontier function need not be continuous in general. We demonstrate that, for a $C^k$-submanifold *M* with $k\ge 2$, the projection map is $C^{k-1}$ on ${\mathscr {E}}(M)$, and we obtain a differentiation formula for the projection map which is used to discuss boundedness of its higher order differentials on tubular neighborhoods. A sufficient condition for the inclusion $M\subseteq {\mathscr {E}}(M)$ is that *M* is a $C^1$ submanifold whose tangent spaces satisfy a local Lipschitz condition. We prove in a new way that this condition is also necessary. More precisely, if *M* is a topological submanifold with $M\subseteq {\mathscr {E}}(M)$, then *M* must be $C^1$ and its tangent spaces satisfy the same local Lipschitz condition. A final section is devoted to highlighting some relations between ${\mathscr {E}}(M)$ and the topological skeleton of $M^c$.

## Introduction

In many problems from analysis and numerical analysis it is important to know the regularity of the orthogonal projection *p* onto a sufficiently regular submanifold *M* of ${{\mathbb {R}}}^d$, that is, the function which maps points from the ambient space of *M* to their nearest element on *M*, if such an element exists uniquely, as well as the regularity of the respective distance function (see Definition 1 for a precise definition). A classical example is the Dirichlet problem for quasilinear partial differential equations, where the manifold of interest is the boundary of the underlying domain [8, 20]. The regularity properties of the distance function are crucial in the Lemma of Hopf, see Lions [14] and also Hopf [10]. Another more recent instance is the study of stochastic differential equations with discontinuous drift and their numerical solvability in Leobacher and Szölgyenyi [12, 13] or Neuenkirch et al. [16], where the manifold of interest is the set of discontinuities of the drift coefficient.

Early results are limited to the restriction of *p* to a small neighborhood of *M*, such as the tubular neighborhood theorems in Hirsch [9], Federer [6, 7], or Krantz and Parks [11]. The first non-local result we could find is contained in the article of Dudek and Holly [5], where the regularity of the distance function and the orthogonal projection is studied on the maximal open domain of definition ${\mathscr {E}}(M)$ of the projection function. The manifolds considered by Dudek and Holly are required to be $C^1$ with a local Lipschitz condition on the tangent bundle, referred to as $C^{1,1}$ (see Definition 3). Under these assumptions they showed that $M\subseteq {\mathscr {E}}(M)$ (we restate their result in Theorem 1).

The four major results of our article contribute to an almost complete understanding of the conditions on the submanifold for existence and regularity of the projection map and the shape of its domain, and will be presented subsequently. The first of these results concerns the shape of ${\mathscr {E}}(M)$ for a given submanifold, which can be described by the frontier function $\vartheta$ for *M*. The latter measures locally how far one can move from a point of *M* in orthogonal direction without leaving ${\mathscr {E}}(M)$ (see Definition 5), thus quantifying the relation $M\subseteq {\mathscr {E}}(M)$.

### **Theorem A**

*Let* $M\subseteq {{\mathbb {R}}}^d$ *be a* $C^{1,1}$ *submanifold*. *Then*

1. *The frontier function* $\vartheta$ *is lower semi-continuous*;
2. *If* *M* *is* $C^2$, *then* $\vartheta$ *is continuous*.

One important consequence of Theorem A is Corollary 2, which states that ${\mathscr {E}}(M)$ is a fiber bundle if *M* is $C^2$. A counterexample, Example 3, shows that if *M* is $C^{1,1}$ but not $C^2$, then, in general, it only admits a lower semi-continuous frontier function.

The second major result is that the Lipschitz condition on the tangent bundle used by Dudek and Holly [5] is not only sufficient, but even necessary. More precisely, we show in Theorem B that a topological submanifold which satisfies $M\subseteq {\mathscr {E}}(M)$ is necessarily $C^{1,1}$.

### **Theorem B**

*Let* *M* *be a* $C^2$ *submanifold*. *For every* $x\in {\mathscr {E}}(M)\setminus M$ *the differential of* *p* *in* *x* *is given by*

$$\begin{aligned} Dp(x)&=\left ({\text {id}}_{T_{p(x)}(M)}-\Vert x-p(x)\Vert L_{p(x),v}\right )^{-1}P_{T_{p(x)}(M)}, \end{aligned}$$

*where* $v=\Vert x-p(x)\Vert ^{-1}(x-p(x))$ *and* $L_{p(x),v}$ *is the shape operator in direction* *v* at *p*(*x*). *For every* $x\in M$ *the differential of* *p* *in* *x* *is* $Dp(x) =P_{T_{p(x)}(M)}.$

Theorem B has already been proven in Lytchak [15] for submanifolds of Riemannian manifolds. The proof uses methods and results from ${\text {CAT}}(k)$ space theory. Using another method of proof, Rataj and Zajíček [19] provide a proof of Theorem B for submanifolds of ${{\mathbb {R}}}^d$. We provide a new self-contained proof which is a nice application of the Borsuk–Ulam theorem (entering through an elegant proof of Lemma 8) and the Brouwer fixed-point theorem (entering through Lemma 3). We point out in Remark 9 that Theorem B generalizes one direction of the celebrated Blaschke’s Rolling Theorem.

We turn to our third major result: Dudek and Holly [5] show that the projection map is $(k-1)$-times differentiable if the submanifold is of class $C^k$, $k\ge 2$, thus generalizing the local result by Foote [7]. Their result is restated here in Theorem 2. We give a different proof and derive a differentiation formula, which in similar form appeared in the literature, see, e.g., Ambrosio and Mantegazza [1, Section 3]. For an implicit form see Rataj and Zähle [18, Theorem 4.23]. The formula for the differential of *p* in Theorem C needs explaining: When we compose linear maps, we omit the ‘ $\circ$ ’ (like for matrix multiplication). This convention is used throughout the paper.

### **Theorem C**

*Let* *M* *be a* $C^2$ *submanifold*. *For every* $x\in {\mathscr {E}}(M)\setminus M$ *the differential of* *p* *in* *x* *is given by*

$$\begin{aligned} Dp(x)&=\left ({\text {id}}_{T_{p(x)}(M)}-\Vert x-p(x)\Vert L_{p(x),v}\right )^{-1}P_{T_{p(x)}(M)}, \end{aligned}$$

*where* $v=\Vert x-p(x)\Vert ^{-1}(x-p(x))$ *and* $L_{p(x),v}$ *is the shape operator in direction* *v* *at* *p*(*x*). *For every* $x\in M$ *the differential of* *p* *in* *x* *is* $Dp(x) =P_{T_{p(x)}(M)}.$

From this particular formula for the differential we obtain criteria for the boundedness of higher order differentials of the projection, see Corollary 4, which weakens the requirements on the hypersurfaces appearing in [12, 13, 16]. The statement of this corollary uses the concept of a subset’s reach introduced in Federer [6]. Theorem 6 highlights connections between the reach of *M* and its frontier function.

Our final major result is another continuity result for the frontier function of *M*, which depends on the topological skeleton ${\mathscr {S}}(M^c)$ of $M^c$, i.e., the centers of the maximal balls contained in the complement of *M* (cf. Definition 9).

### **Theorem D**

*If* *M* *is a* $C^{1,1}$ *submanifold and* ${\mathscr {S}}(M^c)$ *is closed*, *then* $\vartheta$ *is continuous*.

The paper is organized as follows: In Sect. 2 we recall and prove basic properties of the projection *p* and the set ${\mathscr {E}}(M)$. Some of these results are of independent interest as they also hold for general subsets $M\subseteq {{\mathbb {R}}}^d$. The section also contains Theorem A and its corollaries. In Sect. 3 we prove regularity of *p* and the corresponding distance function (Theorem 2) and give a differentiation formula for *p* in terms of the manifold’s shape operator (Theorem C). We relate the boundedness of the (higher order) differentials of unit normal fields of a $C^k$ hypersurface, $k\ge 2$, with positive reach to the boundedness of the higher order differentials of *p* (Corollary 4). Section 4 is dedicated to the proof of Theorem B and finally, Sect. 5 highlights the relation between ${\mathscr {E}}(M)$ and the medial axis/topological skeleton of $M^c$ in Theorem 8. It contains a version of the medial axis transform adapted to our setting as well as Theorem D.

## Parametrization of ${\mathscr {E}}(M)$

We give some basic definitions and introduce some notation used throughout the paper.

### **Definition 1**

Let $M\subseteq {{\mathbb {R}}}^d$ be a nonempty set.

1. For every point $x\in {{\mathbb {R}}}^d$ denote the distance between *x* and *M* by $d(x,M):=\inf \{\Vert x-\xi \Vert :\xi \in M\}$, where $\Vert \cdot \Vert$ is the Euclidean norm on ${{\mathbb {R}}}^d$.
  We denote the distance function $\delta _M:{{\mathbb {R}}}^d\rightarrow {[0,\infty )}$ by $\delta _M(x)=d(x,M)$.
2. For $\varepsilon \in (0,\infty )$, we denote the $\varepsilon$ *-neighborhood* of *M* by
  $$\begin{aligned} M^\varepsilon :=\{x\in {{\mathbb {R}}}^d: d(x,M)<\varepsilon \}\, . \end{aligned}$$
3. We define
  $$\begin{aligned} {\text {unpp}}(M):=\{x\in {{\mathbb {R}}}^d :\,\exists !\, \xi \in M : \Vert x-\xi \Vert =d(x,M)\}\ . \end{aligned}$$
  Thus there exists $p: {\text {unpp}}(M)\rightarrow M$ such that for all $x\in {\text {unpp}}(M)$ it holds that *p*(*x*) is the unique nearest point to *x* on *M*. The function *p* is called the (orthogonal) projection onto *M*.
  A set $U\subseteq {{\mathbb {R}}}^d$ has the *unique nearest point property (unpp) with respect to M* iff $U\subseteq {\text {unpp}}(M)$.
4. Let ${\mathscr {E}}(M):=\bigcup \{U \subseteq {{\mathbb {R}}}^d:U \text { is open and }U\subseteq {\text {unpp}}(M)\}={\text {unpp}}(M)^\circ$ be the maximal open set on which the function *p* is defined.

### **Notation**

*(Balls)* For $x\in {{\mathbb {R}}}^d$ and $r\in (0,\infty )$ denote by

$$\begin{aligned} B_r(x)&:=\{z\in {{\mathbb {R}}}^d:\Vert x-z\Vert <r\} \end{aligned}$$

the open ball with center *x* and radius *r* and by

$$\begin{aligned} {\bar{B}}_r(x)&:=\{z\in {{\mathbb {R}}}^d:\Vert x-z\Vert \le r\}=\overline{B_r(x)} \end{aligned}$$

the closed ball. We denote the $(d-1)$-dimensional unit sphere by $S:={\bar{B}}_1(0)\setminus B_1(0)$.

### **Notation**

*(Line segments)* For $x_1,x_2\in {{\mathbb {R}}}^d$ denote

$$\begin{aligned} {]}x_1,x_2{[}&:=\{(1-\lambda )x_1+\lambda x_2:\lambda \in (0,1)\} \end{aligned}$$

and let ${]}x_1,x_2],[x_1,x_2{[},[x_1,x_2]$ be the corresponding sets with (0, 1) replaced by (0, 1], [0, 1), [0, 1], respectively.

### **Definition 2**

Let $d,m,k\in {{\mathbb {N}}}\cup \{0\}$, $m < d$, and let $M\subseteq {{\mathbb {R}}}^d$. We say *M* is an *m*-dimensional $C^k$ *submanifold* of ${{\mathbb {R}}}^d$ iff for every $\xi \in M$ there exist open sets $U,V\subseteq {{\mathbb {R}}}^d$ and a $C^k$ diffeomorphism $\varPsi :V\rightarrow U$ such that $\xi \in U$ and for all $y=(y_1,\ldots ,y_d)\in V$ it holds $\varPsi (y)\in M \Longleftrightarrow y_{m+1}=\cdots =y_d=0$. In the case where $k=0$, by a $C^0$ diffeomorphism we mean a homeomorphism, and we also call *M* a *topological submanifold*. For the case $k\ge 1$, we write $T_\xi (M)$ for the tangent space on *M* in $\xi \in M$,

$$\begin{aligned} T_\xi (M):=\{t\in {{\mathbb {R}}}^d :\exists \gamma :(-1,1)\rightarrow M \text { a} C^1 \text {map with }\gamma (0)=\xi \text { and }\gamma '(0)=t \}. \end{aligned}$$

### *Remark 1*

Usually, the case $m=d$ is not excluded in the definition of a submanifold. However, for the questions considered here this case is not very interesting: a *d*-dimensional submanifold $M\subseteq {{\mathbb {R}}}^d$ is an open subset of ${{\mathbb {R}}}^d$. Thus, no point in ${{\mathbb {R}}}^d\setminus M$ has a nearest point on *M* and for every $x\in M$ we have $p(x)=x$, so ${\mathscr {E}}(M)=M$ and *p* is $C^\infty$ on ${\mathscr {E}}(M)$.

This means that the case $m=d$ is not interesting for the kind of questions we pursue in this article, and we shall always assume $m<d$.

### *Remark 2*

1. If *M* is a $C^k$ submanifold with $k\ge 0$, $\xi \in M$ and $\varPsi :V\rightarrow U$ is a diffeomorphism as in Definition 2, then the map $\psi :\{y\in {{\mathbb {R}}}^m:(y_1,\ldots ,y_m,0,\ldots ,0)\in V\}\rightarrow {{\mathbb {R}}}^d$,
  $$\begin{aligned} \psi (y_1,\ldots ,y_m):=\varPsi (y_1,\ldots ,y_m,0,\ldots ,0) \end{aligned}$$
  is a (local) parametrization of *M* with $\xi$ in its image. We may always assume that $0\in V$ and $\xi =\psi (0)$.
2. If *M* is $C^1$ and $\xi \in M$ then, by virtue of the implicit function theorem, *M* can be locally represented as the graph of a $C^1$ function $\varPhi$ from the tangent space $T_\xi (M)$ into the corresponding normal space $T_\xi (M)^\perp$.
  More precisely, there exist open sets $W\subseteq T_\xi (M)$ and $U\subseteq T_\xi (M)^\perp$ with $0\in W\cap U$ and a $C^1$ function $\varPhi :W\rightarrow U$ such that $\varPhi (0)=0$ and $M\cap (\xi +W+U)=\{\xi +t+\varPhi (t):t\in W\}$.
  One cannot generalize the statement to topological submanifolds, even if the tangent space is replaced by some other linear space: consider as a counterexample $M:=\{x^2:x\in [0,\frac{1}{2})\}\cup \{x^3:x\in [0,\frac{1}{2})\}$.

The following definition corresponds to condition (3.3) in [5].

### **Definition 3**

Let $m\in {{\mathbb {N}}}\setminus \{0\}$. Denote by $G(m,{{\mathbb {R}}}^d)$ the Grassmannian of *m*-dimensional subspaces of ${{\mathbb {R}}}^d$. For $T_1,T_2\in G(m,{{\mathbb {R}}}^d)$ their *Hausdorff distance* is defined as

$$\begin{aligned} d_H(T_1,T_2):=\sup \left \{\inf \{\Vert t_2-t_1\Vert :t_2\in T_2\cap S\}:t_1\in T_1\cap S\right \}. \end{aligned}$$

We say *M* is an *m*-dimensional $C^{1,1}$ *submanifold* of ${{\mathbb {R}}}^d$ iff *M* is an *m*-dimensional $C^1$ submanifold of ${{\mathbb {R}}}^d$ and the map $M\rightarrow G(m,{{\mathbb {R}}}^d)$, $\xi \mapsto T_\xi (M)$ is locally Lipschitz w.r.t. the Hausdorff distance, i.e., if for all $\xi \in M$ there exists an open set $V\subseteq M$ and a positive constant *L* such that $\xi \in V$ and $\forall \eta \in V:d_H\left (T_\xi (M),T_\eta (M)\right )\le L \Vert \xi -\eta \Vert$.

### **Definition 4**

Let $k\ge 1$. For a $C^k$ submanifold *M* of ${{\mathbb {R}}}^d$ let

$$\begin{aligned} \nu (M):=\{(\xi ,v)\in {{\mathbb {R}}}^d\times {{\mathbb {R}}}^d:\xi \in M,v\perp T_\xi (M)\} \end{aligned}$$

be the *normal bundle* for *M*. Moreover, let

$$\begin{aligned} \nu _1(M):=\{(\xi ,v)\in \nu (M):\Vert v\Vert =1\} \end{aligned}$$

and define the *endpoint map* $F:\nu (M)\rightarrow {{\mathbb {R}}}^d$ by $F(\xi ,v):=\xi +v$.

### *Remark 3*

As is stated in [7], if *M* is an *m*-dimensional $C^k$ submanifold of ${{\mathbb {R}}}^d$, then $\nu (M)$ is a $m+(d-m)$-dimensional $C^{k-1}$ submanifold of ${{\mathbb {R}}}^d\times {{\mathbb {R}}}^d$. We include an explanation of this in the “Appendix”. It follows that *F* is a $C^{k-1}$ function.

The following result is a direct consequence of Dudek and Holly [5, Theorem 3.8].

### **Theorem 1**

*Let* $M \subseteq {{\mathbb {R}}}^d$ *be a* $C^{1,1}$ *submanifold and let* $\xi \in M$. *Then* $\xi$ *has an open neighborhood* *U* *in* ${{\mathbb {R}}}^d$ *so that* $U\subseteq {\text {unpp}}(M)$ *and for all* $x\in U$, $\zeta \in U\cap M$ *with* $x-\zeta \perp T_\zeta (M)$ it holds $p(x)=\zeta$, i.e., $p(F(\zeta ,x-\zeta ))=\zeta$.

### *Remark 4*

Note that Theorem 1 implies that $M\subseteq {\mathscr {E}}(M)$ if *M* is $C^{1,1}$.

Note further that if $x\in {\mathscr {E}}(M)\setminus M$, then $(x-p(x))\perp T_{p(x)}(M)$, since the sphere ${\bar{B}}_{x-p(x)}(x)\setminus B_{x-p(x)}(x)$ has a first-order contact with *M* in *p*(*x*).

The next lemma shows that unpp cannot be a property of isolated points. The lemma is further strengthened by Lemmas 2 and 3.

### **Lemma 1**

*Let* $M\subseteq {{\mathbb {R}}}^d$, $x\in {{\mathbb {R}}}^d\setminus M$, *and assume that there exists* $\xi \in M$ *with* $\Vert x-\xi \Vert =d(x,M)$.

*Then the line segment* ${]}x,\xi ]$ *has the unpp w.r.t. M*, *i.e*., ${]}x,\xi ]\subseteq {\text {unpp}}(M)$, *and for every* $z\in {]}x,\xi ]$ *it holds that* $p(z)=\xi$.

### *Proof*

Let $z\in {]}x,\xi ]$. For $z=\xi$ the assertion is obvious. Now consider the case $z\ne \xi$, and let $\eta \in {\overline{M}}$. We have

1 $$\begin{aligned} \Vert x-\xi \Vert&=\Vert x-z\Vert +\Vert z-\xi \Vert \nonumber \\ \Vert x-\eta \Vert&\le \Vert x-z\Vert +\Vert z-\eta \Vert . \end{aligned}$$

By the continuity of the distance function we have $\Vert x-\xi \Vert \le \Vert x-\eta \Vert$, so that $\Vert z-\xi \Vert \le \Vert z-\eta \Vert$.

Suppose $\Vert z-\xi \Vert =\Vert z-\eta \Vert$. Thus equality holds in (1), which implies $z\in [x,\eta ]$. $z\ne x$ by assumption and $z\ne \eta$ because, $\Vert z-\eta \Vert =\Vert z-\xi \Vert >0$. Thus $z=\lambda \eta +(1-\lambda )x$ for some $\lambda \in (0,1)$. On the other hand $z=\mu \xi +(1-\mu )x$ for some $\mu \in (0,1)$, so that

$$\begin{aligned} \eta -z=\frac{1-\lambda }{\lambda }(z-x)\quad \text { and }\quad \xi -z=\frac{1-\mu }{\mu }(z-x). \end{aligned}$$

From $\Vert z-\xi \Vert =\Vert z-\eta \Vert$ we conclude $\lambda =\mu$ and thus $\eta =\xi$. $\square$

In [6, Theorem 4.8] it is shown that for every closed set *M* the projection map *p* onto *M* is continuous on every set where it is well-defined. The subsequent proposition is a version for which closedness is not needed; for every subset $M\subseteq {{\mathbb {R}}}^d$ the projection *p* is continuous on every *open* set on which it is well-defined. The following result can be found in [5, Theorem 1.3].

### **Proposition 1**

(Continuity of *p*) *Let* $M\subseteq {{\mathbb {R}}}^d$, $U\subseteq {{\mathbb {R}}}^d$ *be an open set with* $U\subseteq {\text {unpp}}(M)$, *and let* $p :U\rightarrow M$ *denote the corresponding projection map*. *Then* *p* *is continuous*.

### *Example 1*

Consider the following example of a non-compact submanifold: 
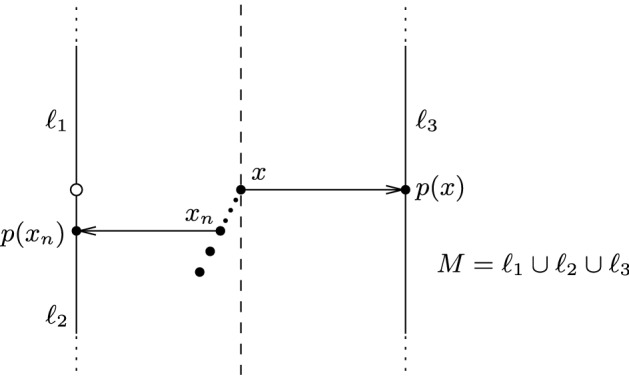


Here the projection is not continuous in *x*. On the other hand, there is no open set *U* containing *x* and having the unpp.

Also the next result can be found in [5, Theorem 1.5.(ii)].

### **Lemma 2**

*Let* $M\subseteq {{\mathbb {R}}}^d$ *and* $x\in {{\mathbb {R}}}^d\setminus M$ *such that there exists an open set* $U\subseteq {\text {unpp}}(M)$ *containing* *x*. *Then there exists an open set* ${\hat{U}}\subseteq {\text {unpp}}(M)$ *that contains* ]*x*, *p*(*x*)[.

### **Lemma 3**

*Let* $M\subseteq {{\mathbb {R}}}^d$ *and* $x\in {{\mathbb {R}}}^d\setminus M$ *such that there exists an open set* $U\subseteq {\text {unpp}}(M)$ *containing* *x*. *Then there exists* $a\in (1,\infty )$ *such that*

$$\begin{aligned} p(x)+a(x-p(x))\in U \quad {\text {and}}\quad p(p(x)+a(x-p(x)))=p(x). \end{aligned}$$

### *Proof*

Consider a closed ball $B:={\bar{B}}_\varepsilon (p(x))$ with $\varepsilon \le \frac{\sqrt{3}}{2}\Vert x-p(x)\Vert$. Then for every $y\in B$ it holds that the (unsigned) angle ${\sphericalangle }(x-y,x-p(x))$ lies in the interval $[0,\frac{\pi }{3}]$. By the continuity of *p* (Proposition 1) there exists $\delta \in (0,\Vert x-p(x)\Vert -\varepsilon )$ such that $\forall z\in {{\mathbb {R}}}^d:\Vert z-x\Vert \le \delta \Rightarrow \left (z\in U \text { and } p(z)\in B\right )$. Let

$$\begin{aligned} {\mathscr {D}}&:=\left \{z\in {{\mathbb {R}}}^d:\exists v \in {{\mathbb {R}}}^d:z=x+\tfrac{1}{2}\delta \Vert x-p(x)\Vert ^{-1}(x-p(x))+v,\right.\\&\qquad \left.\langle x-p(x), v\rangle =0 \quad \text { and }\quad \Vert x-z\Vert \le \delta \right \}, \end{aligned}$$

i.e., ${\mathscr {D}}$ is the $(d-1)$-dimensional closed ball which is orthogonal to $x-p(x)$, lies on the side of *x* opposing *p*(*x*), and has the property that, for all $z\in {\mathscr {D}}$, the angle ${\sphericalangle }(z-x,x-p(x))$ lies in the interval $[0,\frac{\pi }{3}]$. 
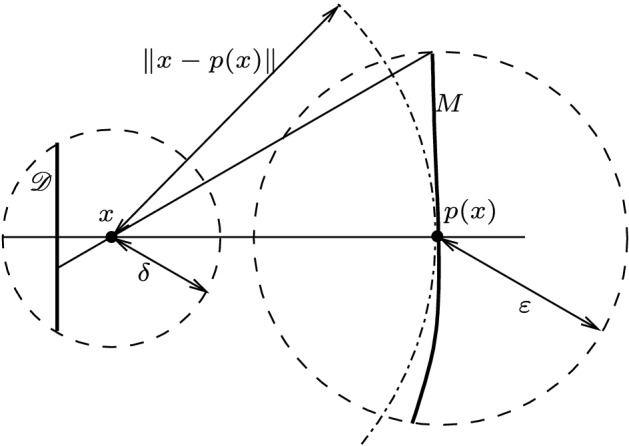


Therefore, every line spanned by $y\in B$ and *x* has precisely one intersection with ${\mathscr {D}}$. Define this as *f*(*y*) and consider the mapping $g:B\rightarrow B$ defined by $g(y):=p(f(y))$. Note that *f* is continuous, so *g* is a continuous mapping from $B\rightarrow B$. Since *B* is homeomorphic to the unit ball in ${{\mathbb {R}}}^d$, there exists a fixed point $y_0$ of *g* by Brouwer’s fixed point theorem, i.e., there exists $y_0\in B$ such that $g(y_0)=y_0$. Note that $y_0\in M$ by the definition of *g*.

Now $p(f(y_0))=y_0$ and $x\in {]}f(y_0),y_0{[}$. By Lemma 1 we therefore have $p(x)=y_0$ and thus $p(f(y_0))=p(x)$.

We conclude the proof by noting that $f(y_0)$ is of the desired form $f(y_0)=p(x)+a(x-p(x))$ with $a\in (1,\infty )$. $\square$

### *Remark 5*

Let us revisit Example 1. The point *x* lies in ${\text {unpp}}(M)$ and $]x,p(x)[\subseteq {\mathscr {E}}(M)$. However, $x\notin {\mathscr {E}}(M)$, and also there does not exist $a\in (1,\infty )$ such that $p(p(x)+a(x-p(x)))=p(x)$. Therefore, the assumption made in Lemma 3, that *x* be contained in some open set in ${\text {unpp}}(M)$, is necessary.

### *Example 2*

Consider the set $M:=\{(x,x^2)\in {{\mathbb {R}}}^2:x\ge 0\}$. Routine calculations yield that

$$\begin{aligned} {\mathscr {E}}(M)&={{\mathbb {R}}}^2\setminus \left \{(x,y):x\le 0,\ y=\tfrac{1+3(x^2)^\frac{1}{3}}{2}\right \},\\ {\text {unpp}}(M)&={\mathscr {E}}(M)\cup \{(0,\tfrac{1}{2})\}. \end{aligned}$$

The point $\left( 0,\frac{1}{2}\right)$ has a unique closest point on *M*, namely (0, 0), but does not lie in ${\mathscr {E}}(M)$. Of course, for all $y\in [0,\frac{1}{2}]$ we have $p\left ((0,y)\right )=(0,0)=p\left ((0,\frac{1}{2})\right )$, but for $y\in  [\frac{1}{2},\infty )$ it holds $p\left ((0,y)\right )=\left (\sqrt{\frac{2y-1}{4}},\frac{2y-1}{4}\right )$.

In this example *p* is continuous on ${\text {unpp}}(M)\ne {\mathscr {E}}(M)$, but still the conclusion of Lemma 3 does not hold since there is no open set $U\subseteq {\text {unpp}}(M)$ with $(0,\frac{1}{2})\in U$.

### **Definition 5**

Let *M* be a $C^{1,1}$ submanifold of ${{\mathbb {R}}}^d$.

1. We define
  $$\begin{aligned} \nu ^*(M)&:=\left \{(\xi ,v)\in \nu (M):\left [\xi ,\xi +v\right ]\subseteq {\mathscr {E}}(M)\right \}, \end{aligned}$$
  and $F^*:\nu ^*(M)\rightarrow {\mathscr {E}}(M)$ by $F^*(\xi ,v):=\xi +v$, the restriction of the endpoint map to $\nu ^*(M)$.
2. We define $\vartheta :\nu _1(M)\rightarrow (0,\infty ],$ $\vartheta (\xi ,v):=\sup \{r> 0:{]}\xi ,\xi +rv{[}\subseteq {\mathscr {E}}(M)\}$ and call $\vartheta$ the *frontier function* for *M*.

### **Proposition 2**

*Let* *M* *be a* $C^{1,1}$ *submanifold of* ${{\mathbb {R}}}^d$. *Then*

1. *The frontier function* $\vartheta$ *is well-defined*,
2. *For all* $(\xi ,v)\in \nu _1(M)$ *and all* $r\in \big [0,\vartheta (\xi ,v)\big )$ *it holds* $p(\xi +rv)=\xi$,
3. $$ \nu ^*(M)=\big \{(\xi ,rv):(\xi ,v)\in \nu _1(M), r\in \big [0,\vartheta (\xi ,v)\big )\big \}, $$
4. *For every* $x\in {\mathscr {E}}(M)$ *we have* $[x,p(x)]\subseteq {\mathscr {E}}(M)$.

### *Proof*

1. By Theorem 1, for all $\xi \in M$ there exists an open set $U\subseteq {\text {unpp}}(M)$ containing $\xi$. Since *U* is open and $U\subseteq {\mathscr {E}}(M)$, the set $\big \{r\in (0,\infty ):{]}\xi ,\xi +rv{[}\subseteq {\mathscr {E}}(M)\big \}$ is non-empty and therefore $\vartheta (\xi ,v)>0$ for all $(\xi ,v)\in \nu _1(M)$.
2. Clearly $p(\xi )=\xi$. Now let $H:=\big \{r\in (0,\infty ):p(\xi +r v)=\xi \big \}$. The set *H* is non-empty by Theorem 1, so $s:=\sup H$ is a positive real number or equal to infinity. Assume $s<\vartheta (\xi ,v)$. Then $\xi +s v\in {\big ]\xi ,\xi +\frac{1}{2}(s+\vartheta (\xi ,v))v\big [}\subseteq {\mathscr {E}}(M)$, in particular $\xi +s v\in {\mathscr {E}}(M)$. Since $p(\xi +rv)=\xi$ for all $r\in (0,s)$ and *p* is continuous on ${\mathscr {E}}(M)$, it holds $p(\xi +s v)=\xi$. By Lemma 3 there exists $a\in (1,\infty )$ such that
  $$\begin{aligned} \xi + asv\in {\mathscr {E}}(M) \text { and }p\big (\xi + asv\big )=\xi . \end{aligned}$$
  Hence, by Lemma 2 also $]\xi , \xi + asv[\subseteq {\mathscr {E}}(M)$, contradicting $s:=\sup H$. Therefore, $s\ge \vartheta (\xi ,v)$ and $p(\xi +r v)=\xi$ for all $r\in (0,\vartheta (\xi ,v))$.
3. This is obvious.
4. By Lemma 2 we have that for every $x\in {\mathscr {E}}(M)$ also the line segment [*x*, *p*(*x*)[ is contained in ${\mathscr {E}}(M)$. By Theorem 1 we have $M\subseteq {\mathscr {E}}(M)$ so that indeed $[x,p(x)]\subseteq {\mathscr {E}}(M)$.

$$\square $$

### **Lemma 4**

*Let* *M* *be a* $C^{1,1}$ *submanifold*. *Then* $F^*$ *is a homeomorphism and* $p(F^*(\xi ,v))=\xi$ *for all* $(\xi ,v)\in \nu ^*(M)$.

### *Proof*

1. $F^*$ is injective: Let $(\xi ,v),(\zeta ,w)\in \nu ^*(M)$ with $\xi +v=F^*(\xi ,v)=F^*(\zeta ,w)=\zeta +w$. From item 2 of Proposition 2 it follows $p(\xi +v)=\xi$ and $p(\zeta +w)=\zeta$. By the unpp of ${\mathscr {E}}(M)$, it holds $\xi =p(\xi +v)=p(\zeta +w)=\zeta$. Together with $\xi +v=\zeta +w$ we also get $v=w$.
2. $F^*$ is surjective: since every $x\in {\mathscr {E}}(M)$ can by written as $x=p(x)+(x-p(x))$ and $(x-p(x))\perp T_{p(x)}(M)$ (see Remark 4), we have $x=F^*(p(x),x-p(x))$. By Proposition 2.(4), ${[p(x),x]}\subseteq {\mathscr {E}}(M)$.
3. The function $F^*$ is clearly continuous. Its inverse satisfies $(F^*)^{-1}(x)=(p(x),x-p(x))$, and it is continuous since *p* is continuous by Proposition 1.

$$\square $$

We recall some well-known concepts.

### **Definition 6**

Let *M* be an *m*-dimensional $C^1$ submanifold of ${{\mathbb {R}}}^d$.

1. Let $V\subseteq M$ be an open set relative to *M* and let $n:V\rightarrow {{\mathbb {R}}}^d$ be a continuous function such that $\Vert n(\eta )\Vert =1$ and $n(\eta )\in (T_\eta (M))^\perp$ for every $\eta \in V$. Then we call $\big (V,n\big )$ a *unit normal field*.
2. Let $V\subseteq M$ be an open set relative to *M* and let $n_{m+1},\ldots ,n_d:V\rightarrow {{\mathbb {R}}}^d$ be continuous functions such that
  $$\begin{aligned} \langle n_j(\eta ), n_\ell (\eta )\rangle = {\left\{ \begin{array}{ll} 1&{} j=\ell \\ 0&{} j\ne \ell \end{array}\right. } \end{aligned}$$
  and $n(\eta )\in (T_\eta (M))^\perp$ for every $\eta \in V$. Then we call $\big (V,n_{m+1},\ldots ,n_d\big )$ an *orthonormal moving frame of* $\nu (V)$.

It is not hard to show—using the subsequent proposition and induction—that if *M* is a $C^k$ submanifold with $k\ge 1$, then for every $\xi \in M$ there exists a $C^{k-1}$ orthonormal moving frame $\big (V,n_{m+1},\ldots ,n_d\big )$ of $\nu (V)$ with $\xi \in V$.

### **Proposition 3**

*Let* *M* *be an* *m*-*dimensional* $C^k$ *submanifold of* ${{\mathbb {R}}}^d$, *with* $k\ge 1$. *Then for every* $(\xi ,v)\in \nu _1(M)$ *there exists a* $C^{k-1}$ *unit normal field* $\big (V,n\big )$ *with* $\xi \in V$ *and* $n(\xi )=v$.

*For* $k\ge 2$ *it holds that*, *if* $(V_1,n_1)$ *is another unit normal field with* $\xi \in V_1$ *and* $n_1(\xi )=v$, *then*

$$\begin{aligned} P_{T_\xi (M)} Dn(\xi )=P_{T_\xi (M)} Dn_1(\xi ), \end{aligned}$$

*where* $P_{T_\xi (M)}$ *is the projection onto the tangent space* $T_\xi (M)$.

### *Proof*

Let $(\xi ,v)\in \nu _1(M)$. Choose a local parametrization $\psi :W\rightarrow {{\mathbb {R}}}^d$ of *M* with $0\in W$ and $\psi (0)=\xi$ (see item 1 of Remark 2). For every $y\in W$ and every $j\in \{1,\ldots ,m\}$ define $t_j(y):=\frac{\partial }{\partial y_j}\psi (y)$. Note that for every $y\in W$ the set $\{t_1(y),\ldots ,t_d(y)\}$ forms a basis of the tangent space $T_{\psi (y)}(M)$.

If $m=d-1$, then the cross product $w:=t_1\times \cdots \times t_d$ is normal to *M* and $w(\xi )=\lambda v$ for some $\lambda \in {{\mathbb {R}}}\setminus \{0\}$. W.l.o.g., $\lambda >0$. Now the vector field $n=\Vert w\Vert ^{-1}w$ is a unit normal field on $V=\psi (W)$ with $\xi \in V$ and $n(\xi )=v$.

Now consider the case $m<d-1$. Write $v_{m+1}:=v$ and extend $v_{m+1}$ to a basis $v_{m+1},v_{m+2},\ldots , v_d$ of $\big (T_{\psi (0)}(M)\big )^\perp$. Then

$$\begin{aligned} \det \big ( t_1(0),\ldots ,t_m(0),v_{m+1},\ldots , v_d\big )\ne 0, \end{aligned}$$

and by the continuity of the determinant and the functions $t_1,\ldots , t_m$ there exists $c\in (0,\infty )$ and an open set $W_1\subseteq W$ containing 0 such that for all $y\in W_1$ we have $\big |\det \big (t_1(y),\ldots ,t_d(y),v_{m+1},\ldots , v_d\big )\big |\ge c.$

Denote by *P*(*y*) the orthogonal projection from ${{\mathbb {R}}}^d$ onto the space spanned by $\{t_1(y),\ldots ,t_m(y)\}$, i.e., on $T_{\psi (y)}(M)$, and define $n(\psi (y))$ by

$$\begin{aligned} n(\psi (y))&:=\Vert v_{m+1}-P(y)v_{m+1}\Vert ^{-1} (v_{m+1}-P(y)v_{m+1}). \end{aligned}$$

Finally, in both cases, $V:=\psi (W_1)$ is an open subset of *M* by the invariance of domain theorem and we have $\xi =\psi (0)\in V$. So (*V*, *n*) is a unit normal field with $\xi \in V$ and $n(\xi )=v$.

Let $(V_1,n_1)$ be another unit normal field with $\xi \in V_1,n_1(\xi )=v$.

$$\begin{aligned} P_{T_\xi (M)}D n(\xi )-P_{T_\xi (M)}D n_1(\xi )&=P_{T_\xi (M)}D (n- n_1)(\xi ). \end{aligned}$$

For every $C^1$ vector field $(V_2,t)$, with $t:V_2\subset V\cap V_1 \rightarrow {{\mathbb {R}}}^d$ satisfying $t(\zeta )\in T_\zeta (M)$ for all $\zeta$, we have

$$\begin{aligned}&\langle n-n_1,t\rangle =0\\&t^\top D (n-n_1)+ (n-n_1)^\top D t=0. \end{aligned}$$

Since $(n-n_1)(\xi )=0$, we have $t^\top D (n-n_1)(\xi )=0$. Because *t* was arbitrary the result follows. $\square$

### **Definition 7**

Let *M* be a $C^1$ submanifold of ${{\mathbb {R}}}^d$ and $(\xi ,v)\in \nu _1(M)$.

1. For an arbitrary $C^1$ unit normal field (*V*, *n*) with $\xi \in V$, $n(\xi )=v$ we define the *shape operator* $L_{\xi ,v}:T_\xi (V)\rightarrow T_\xi (V)$ by
  $$\begin{aligned} L_{\xi ,v}:= -P_{T_\xi (M)} Dn(\xi ), \end{aligned}$$
  where $P_{T_\xi (M)}$ denotes the orthogonal projection onto the tangent space. Note that, $L_{\xi ,v}$ is well-defined by Proposition 3.
2. Denote by $\lambda _1,\ldots ,\lambda _\ell$ the (not necessarily distinct) positive eigenvalues of $L_{\xi ,v}$. Then the points $\xi +\lambda _1^{-1}v,\ldots ,\xi +\lambda _\ell ^{-1} v$ are called *centers of curvature* of *M* in $\xi$ in direction of *v*.
3. For every $(\xi ,v)\in \nu _1(M)$ denote by $\varrho (\xi ,v)$ the *radius of curvature* of *M* at $\xi$ in direction of the unit normal *v*,
  $$\begin{aligned} \varrho (\xi ,v):=\inf \{r\in (0,\infty ):\forall \tau \in (0,\infty ):B_\tau (\xi )\cap M\cap B_r(\xi +rv)\ne \emptyset \}, \end{aligned}$$
  with the convention that $\inf \emptyset =\infty$.

The following fact is most likely folklore, yet it is not easy to find a citation for item (2). Therefore, a proof is provided in the “Appendix”.

### **Proposition 4**

*Let* $M\subseteq {{\mathbb {R}}}^d$ *be a* $C^2$ *submanifold and let* $(\xi ,v)\in \nu _1(M)$. *Then*

1. *The shape operator* $L_{\xi ,v}$ *is self-adjoint*.
2. *Denote by* $\lambda _1,\ldots ,\lambda _m$ *the* (*not necessarily distinct*) *eigenvalues of* $L_{\xi ,v}$.
  *Then*
  $$\begin{aligned} \varrho (\xi ,v)=\big (\max (0,\max (\lambda _1,\ldots ,\lambda _m))\big )^{-1}, \end{aligned}$$
  *with the convention that* $0^{-1}=\infty$.

The next proposition characterizes the critical values of the endpoint map. It is the second example in Section 1.3 in [2] and also follows from [17, 4.1.9 Corollary].

### **Proposition 5**

*Let* $M\subseteq {{\mathbb {R}}}^d$ *be a* $C^2$ *submanifold and let* $(\xi ,v)\in \nu (M)$. *Then* $\det (DF)(\xi ,v)=0$ *iff* $\xi +v$ *is a center of curvature in* $\xi$ *in direction of* $\Vert v\Vert ^{-1}v$.

A similar observation as in the subsequent lemma can be found in [4, Example 9].

### **Lemma 5**

*Let* $M\subseteq {{\mathbb {R}}}^d$ *be a* $C^{1,1}$ *submanifold*. *Then*

$$\begin{aligned} \vartheta (\xi ,v)\le \varrho (\xi ,v) \end{aligned}$$

*for every* $$(\xi ,v)\in \nu _1(M)$$.

### *Proof*

Let $(\xi ,v)\in \nu _1(M)$. In the case $\varrho (\xi ,v)=\infty$ there is nothing to show. In the case $\varrho (\xi ,v)<\infty$ assume instead $\vartheta (\xi ,v)>\varrho (\xi ,v)$. Choose $r_1\in \left( \varrho (\xi ,v) ,\vartheta (\xi ,v)\right)$. Then $M\cap B_{r_1}(\xi +r_1v)\cap B_\tau (\xi )\ne \emptyset$ for all $\tau \in (0,\infty )$. In particular, $M\cap B_{r_1}(\xi +r_1v)\ne \emptyset$, which implies $d(\xi +r_1v,M)< r_1$. On the other hand, by Lemma 4 we have $\xi +r_1 v\in {\mathscr {E}}(M)$ and $p(\xi +r_1 v)=\xi$, and in particular, $d(\xi +r_1v,M)=r_1$. This is a contradiction. $\square$

### **Lemma 6**

*Let* *M* *be a* $C^2$ *submanifold of* ${{\mathbb {R}}}^d$. *Then the map* $F^*:\nu ^*(M)\rightarrow {\mathscr {E}}(M)$ *is a diffeomorphism*.

### *Proof*

We have already established in Lemma 4 that $F^*$ is a homeomorphism. Furthermore, $F^*$ is differentiable (see Remark 3). Thus, it remains to show that the Jacobian of $F^*$ has full rank in every point $(\xi ,v)\in \nu ^*(M)$. By Proposition 5 this could only fail if $F(\xi ,v)$ was a center of curvature of *M*. By Lemma 5 and Theorem 4 however, no center of curvature is contained in ${\mathscr {E}}(M)$. $\square$

### **Theorem A**

*Let* $M\subseteq {{\mathbb {R}}}^d$ *be a* $C^{1,1}$ *submanifold*. Then

1. *The frontier function* $\vartheta$ *is lower semi-continuous*;
2. *If* *M* *is* $C^2$, *then* $\vartheta$ *is continuous*.

### *Proof*

1. $\vartheta$ is lower semi-continuous: Let $(\xi ,v)\in \nu _1(M)$. We first consider the case where $\vartheta (\xi ,v)<\infty$. Let $\varepsilon \in (0,\infty )$ and $r\in (\vartheta (\xi ,v)-\varepsilon ,\vartheta (\xi ,v))$ such that ${\big ]\xi ,\xi +rv\big [}\subseteq {\mathscr {E}}(M)$ and, since ${\mathscr {E}}(M)$ is open, there exists $\rho \in (0,r-\vartheta (\xi ,v)+\varepsilon )$ with $B_\rho (\xi +rv)=\{z\in {{\mathbb {R}}}^d:\Vert z-(\xi +rv)\Vert <\rho \}\subseteq {\mathscr {E}}(M)$. Since *p* is continuous and $p(\xi +rv)=\xi$ by Lemma 4, we can choose $\delta \in (0,\rho )$ such that
  $$\begin{aligned} \forall z\in {\mathscr {E}}(M):\Vert \xi +r v -z\Vert<\delta \Rightarrow \Big \Vert \Big (p(z)+r\tfrac{z-p(z)}{\left\| z-p(z)\right\| }\Big )-(\xi +rv)\Big \Vert <\rho , \end{aligned}$$
  and in particular $\forall z\in {\mathscr {E}}(M):\Vert \xi +r v -z\Vert <\delta \Rightarrow p(z)+r\tfrac{z-p(z)}{\left\| z-p(z)\right\| }\in {\mathscr {E}}(M)$. By the continuity of the endpoint map there exist $\delta _1,\delta _2\in (0,\infty )$ such that for all $(\zeta ,w)\in \nu _1(M)$ it holds
  $$\begin{aligned} \Vert \zeta -\xi \Vert<\delta _1 \quad \text { and }\quad \Vert w-v\Vert<\delta _2 \Rightarrow \Vert \xi +r v -(\zeta +r w)\Vert <\delta \end{aligned}$$
  and therefore
  $$\begin{aligned} \Vert \zeta -\xi \Vert<\delta _1 \quad \text { and }\quad \Vert w-v\Vert <\delta _2 \Rightarrow \vartheta (\zeta ,w)\ge r>\vartheta (\xi ,v)-\varepsilon . \end{aligned}$$
  This shows that $\vartheta$ is lower semi-continuous in $(\xi ,v)$. The proof for the case $\vartheta (\xi ,v)=\infty$ is similar and is left to the reader.
2. $\vartheta$ is upper semi-continuous if *M* is $C^2$. Assume the opposite. Then there exist $(\xi ,v) \in \nu _1(M)$, $\alpha \in (0,\infty )$, and a sequence $(\xi _k,v_k)_{k\in {{\mathbb {N}}}}$ in $\nu _1(M)$ converging to $(\xi ,v)$ with $\vartheta (\xi _k,v_k)\ge \alpha$ for every $k\in {{\mathbb {N}}}$ but $\vartheta (\xi ,v)<\alpha$. Choose a sequence $(r_k)_{k\in {{\mathbb {N}}}}$ in $[\vartheta (\xi ,v),\alpha )$ with $\lim _k r_k=\alpha$. From Lemma 4 it follows that $\xi _k+r_k v_k\in {\mathscr {E}}(M)$ and $p(\xi _k+r_k v_k)=\xi _k$ for every $k\in {{\mathbb {N}}}$. In other words, for every $k\in {{\mathbb {N}}}$ we have $B_r\left( \xi _k+r_k v_k\right) \cap M=\emptyset$ and $\bar{B}_r\left( \xi _k+r_k v_k\right) \cap M=\{\xi _k\}$. Thus, we obtain $B_\alpha \left( \xi +\alpha v\right) \cap M=\emptyset$ and $\bar{B}_\alpha \left( \xi +\alpha v\right) \cap M\supseteq \{\xi \}$. This together with Lemma 1 implies that for all $r\in [\vartheta (\xi ,v),\alpha )$, we have that $\xi$ is the unique nearest point to $\xi +r v$ in ${\overline{M}}$. In particular, for $z:=\xi +\vartheta (\xi ,v)v$ we have $\{z\}\in {\text {unpp}}({\overline{M}})$. Since $z\notin {\mathscr {E}}(M)$ there is a sequence $(u_k)_{k\in {{\mathbb {N}}}}$ converging to *z* such that for every $k\in {{\mathbb {N}}}$ we have either that $u_k$ has no nearest point on *M* or has at least 2 nearest points on *M*. Consider now any sequence $(\zeta _k)_{k\in {{\mathbb {N}}}}$ in ${\overline{M}}$ with $d(u_k,{{\overline{M}}})=\Vert u_k-\zeta _k\Vert$. It is easy to see that $(\zeta _k)$ is bounded, and therefore has a convergent subsequence $(\zeta _{k_j})_{j\in {{\mathbb {N}}}}$. But then $\Vert z-\lim _{j}\zeta _{k_j}\Vert =\lim _{j}\Vert u_{k_j}-\zeta _{k_j}\Vert \le \lim _{j}\Vert u_{k_j}-\xi \Vert =\Vert z-\xi \Vert$, so that $\Vert z-\lim _{j}\zeta _{k_j}\Vert =\Vert z-\xi \Vert$ and therefore $\lim _{j}\zeta _{k_j}=\xi$. Denote now $P_k:=\big \{\zeta \in {{\overline{M}}}:\Vert u_k-\zeta \Vert =d(u_k,{{\overline{M}}})\big \}$. Then it holds
  2 $$\begin{aligned} \forall \varepsilon >0\, \exists N_\varepsilon : \forall k\ge N_\varepsilon :\sup _{\zeta \in P_k}\Vert \zeta -\xi \Vert \le \varepsilon , \end{aligned}$$
  since otherwise one could find a sequence $(\zeta _k)_{k\in {{\mathbb {N}}}}$ with $\zeta _k\in P_k$ having an accumulation point different from $\xi$, which we found to be impossible in the preceding paragraph. It is readily checked that, since *M* is a submanifold, there exists $\varepsilon \in (0,\infty )$ such that ${\bar{B}}_\varepsilon (\xi )\cap M={\bar{B}}_\varepsilon (\xi )\cap {{\overline{M}}}$. From formula (2) it now follows that there exists $N_\varepsilon$ such that for all $k\ge N_\varepsilon$ we have $P_k\subseteq M$. In addition, it follows that for every $k\ge N_\varepsilon$ the point $u_k$ has at least 2 nearest points on *M*. We have so far succeeded, under the assumption that $\vartheta$ is not upper semi-continuous, to show existence of $z\notin {\mathscr {E}}(M)$ and of a sequence $(u_k)_{k\in {{\mathbb {N}}}}$, such that for every $k\in {{\mathbb {N}}}$ there exist $\zeta _k,\eta _k\in M$ with $\zeta _k\ne \eta _k$ and $\Vert u_k-\zeta _k\Vert =d(u_k,M)=\Vert u_k-\eta _k\Vert$. This means that the endpoint map *F* is not injective on any open neighborhood of $(\xi ,\vartheta (\xi ,v)v)$ in $\nu (M)$. It follows from the inverse function theorem that the differential of *F* is singular at $(\xi ,\vartheta (\xi ,v)v)$. By Proposition 5, $F(\xi ,\vartheta (\xi ,v)v)$ is a center of curvature in $\xi$ in direction of *v*. By Theorem 4 and Lemma 5 we get $\vartheta (\xi ,v)=\varrho (\xi ,v)$. Since *M* is $C^2$, the function $\varrho :\nu _1(M)\rightarrow (0,\infty ]$ is continuous. We have already shown the existence of a sequence $(\xi _k,v_k)_{k\in {{\mathbb {N}}}}$ in $\nu _1(M)$ converging to some pair $(\xi ,v) \in \nu _1(M)$ such that $\vartheta (\xi _k,v_k)\ge \alpha$ for every $k\in {{\mathbb {N}}}$ but $\vartheta (\xi ,v)<\alpha$. From Lemma 5 it follows that $\varrho (\xi ,v)=\lim _{k}\varrho (\xi _k,v_k)\ge \lim _{k}\vartheta (\xi _k,v_k)\ge \alpha > \vartheta (\xi ,v)$. This is the desired contradiction.

$$\square $$

### *Remark 6*

The proof of the first assertion of the preceeding theorem may at first sight seem more complicated than necessary. The technicalities arise since, for example, one cannot simply conclude $\vartheta (\zeta ,w)\ge \beta$ from $B_\beta (\zeta +\beta w)\cap M=\emptyset$.

### *Example 3*

We construct an example of a 1-dimensional submanifold *M* of ${{\mathbb {R}}}^2$ which is $C^{1,1}$ but for which $\vartheta$ is not continuous. *M* is defined as the graph of a function $f:{{\mathbb {R}}}\rightarrow {{\mathbb {R}}}$ with $f(x)=\int _0^x g(y)dy$ and $g:{{\mathbb {R}}}\rightarrow {{\mathbb {R}}}$ is defined as

$$\begin{aligned} g(x):=\left\{ \begin{array}{lll} 0&\quad \text {if }\; x\le 0 \text { or }x>1\\ x-1&\quad \text {if }\;2/3<x\le 1\\ 3^{-(2k+1)}-x&\quad \text {if }\;2\cdot 3^{-2(k+1)}<x\le 2\cdot 3^{-(2k+1)}&\quad \text {for some } k\in {{\mathbb {N}}}\cup \{0\}\\ x-3^{-2k}&\quad \text {if }\;2\cdot 3^{-(2k+1)}<x\le 2\cdot 3^{-2k}&\quad \text {for some } k\in {{\mathbb {N}}}\cup \{0\}. \end{array}\right. \end{aligned}$$

The function *g* is clearly Lipschitz with Lipschitz constant equal to 1 and $T_{(x,f(x))}=\{a (1,g(x)):a\in {{\mathbb {R}}}\}$, so *M* is $C^{1,1}$. It is readily checked that $|g(x)|\le \frac{|x|}{2}$, so $f(x)\le \frac{x^2}{4}$. Since $f(x)=0$ for $x<0$ we have $\vartheta ((x,0),(0,1))\ge 2$ for all $x<0$.

On the intervals $[2\cdot 3^{-(2k+1)}, 2\cdot 3^{-2k}]$ we have $f(x)=(x-3^{-2k})^2+f(3^{-2k})$, such that using also Lemma 5

$$\begin{aligned} \vartheta \big ((3^{-2k},f(3^{-2k}),(0,1)\big )\le \varrho \big ((3^{-2k},f(3^{-2k}),(0,1)\big )=\frac{1}{2}. \end{aligned}$$

So we have found a sequence $(\xi _k,v_k)\in \nu _1(M)$ with $\lim _{k\rightarrow \infty }(\xi _k,v_k)=((0,0),(0,1))$ and $\vartheta (\xi _k,v_k) \le \frac{1}{2}<2\le \lim _{x\nearrow 0}\vartheta \big ((x,0),(0,1)\big )$.

For illustration we plot the graphs of $f'$ and *f*: 
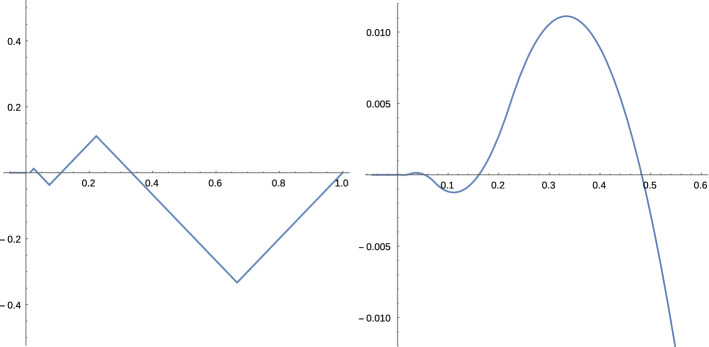


### *Example 4*

Let $$M=\big ([1,\infty )\times \{0\}\big )\cup \{(x_1,x_2)\in {{\mathbb {R}}}^2:(x_1-1)^2+(x_2-1)^2=1,x_1<1,x_2<1\}\cup \big (\{0\}\times [1,\infty )\big )$$.

Obviously, *M* is not $C^2$, but it is readily checked that *M* is $C^{1,1}$ and that $\vartheta$ is continuous.

### *Example 5*

In general, continuity of $\vartheta$ is all we get, even if *M* is $C^\infty$: it is easy to construct examples of $C^\infty$-submanifolds *M* such that $\vartheta$ is not differentiable. We provide a drawing: 
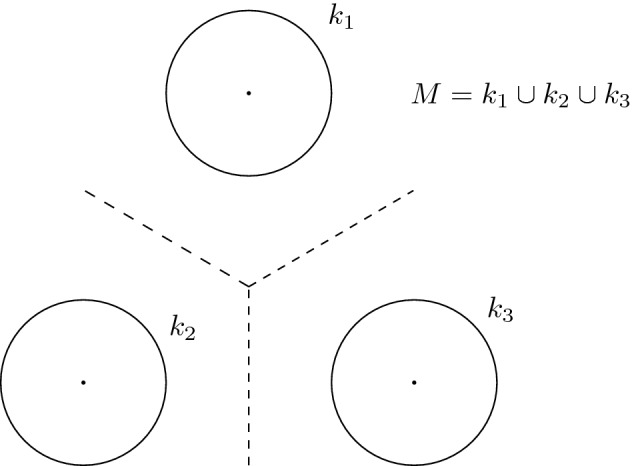


We give two corollaries of Theorem A, the second stating that ${\mathscr {E}}(M)$ is a vector bundle.

### **Corollary 1**

*Let* *M* *be a* $C^2$ *submanifold of* ${{\mathbb {R}}}^d$. *Then the maps*

$$\begin{aligned} {\overline{\vartheta }}:\nu ^*(M)&\rightarrow [1,\infty )\\ (\xi ,v)&\mapsto {\left\{ \begin{array}{ll}\frac{\vartheta \left( \xi ,\frac{v}{\Vert v\Vert }\right) }{\vartheta \left( \xi ,\frac{v}{\Vert v\Vert }\right) -\Vert v\Vert }, &{}\quad {\mathrm{if}}\quad \vartheta \left( \xi ,\frac{v}{\Vert v\Vert }\right)<\infty \quad {\mathrm{and}}\quad v\ne 0,\\ 1 &{}\quad {\mathrm{otherwise}},\end{array}\right. } \\ {\underline{\vartheta }}:\nu (M)&\rightarrow (0,1]\\ (\xi ,w)&\mapsto {\left\{ \begin{array}{ll}\frac{\vartheta \left( \xi ,\frac{w}{\Vert w\Vert }\right) }{\vartheta \left( \xi ,\frac{w}{\Vert w\Vert }\right) +\Vert w\Vert }, &{}\quad {\mathrm{if}}\quad \vartheta \left( \xi ,\frac{w}{\Vert w\Vert }\right) <\infty \quad {\mathrm{and}} w\ne 0,\\ 1 &{}\quad {\mathrm{otherwise}},\end{array}\right. } \end{aligned}$$

*are continuous*.

The normal bundle $\nu (M)$ is a vector bundle. The next corollary states that ${\mathscr {E}}(M)$ is homeomorphic to $\nu (M)$, if *M* is of class $C^2$. Further, the bundle structure naturally transfers from $\nu (M)$ through $\nu ^*(M)$ to ${\mathscr {E}}(M)$.

### **Corollary 2**

*Let* *M* *be a* $C^2$ *submanifold of* ${{\mathbb {R}}}^d$. *Then the map*

$$\begin{aligned} \varphi :\nu ^*(M)&\rightarrow \nu (M)\\ (\xi ,v)&\mapsto (\xi ,{\overline{\vartheta }}(\xi ,v)\,v) \end{aligned}$$

*is a homeomorphism with inverse*

$$\begin{aligned} \varphi ^{-1}:\nu (M)&\rightarrow \nu ^*(M)\\ (\xi ,w)&\mapsto (\xi ,{\underline{\vartheta }}(\xi ,w)\,w). \end{aligned}$$

*Moreover*, *the homeomorphism* $\sigma :=\varphi \circ (F^*)^{-1}:{\mathscr {E}}(M)\rightarrow \nu (M)$ *makes* ${\mathscr {E}}(M)$ *a vector bundle with bundle map* $p:{\mathscr {E}}(M)\rightarrow M$. *Through transport from* $\nu (M)$, *which* is *a vector bundle*, *the vector operations on* $p^{-1}(\{\xi \})$ *for* $\xi \in M$ *are given by* $+_\xi =\sigma ^{-1}\circ +\circ (\sigma \times \sigma )$ *and* $\cdot _\xi =\sigma ^{-1}\circ \cdot \circ ({\mathrm {id}}_{{\mathbb {R}}}\times \sigma )$, *where* $(+,\cdot )$ *are the vector operations on the normal space* $(T_\xi M)^\perp$.

### *Proof*

The first part follows immediately from Corollary 1. For the second part one needs to check the vector space axioms for the vector addition $+_\xi$ and scalar multiplication $\cdot _\xi$ on $p^{-1}(\{\xi \})$, as well as the fiber bundle compatibility condition. The former follows by structure transport from $(T_\xi M)^\perp$, the latter is a consequence of the fact that $\varphi$ is a homeomorphism. $\square$

Consider the following concept which has first been defined in [6].

### **Definition 8**

Let $M\subseteq {{\mathbb {R}}}^d$. The *reach* of *M* is the largest $\varepsilon _0$ (if it exists) such that $M^{\varepsilon _0}\subseteq {\text {unpp}}(M)$, i.e.,

$$\begin{aligned} {\text {reach}}(M)=\sup \{\varepsilon \in (0,\infty ):M^\varepsilon \subseteq {\text {unpp}}(M)\}. \end{aligned}$$

Note that ${\text {reach}}(M)\in [0,\infty ]$. We call *M* a set of *positive reach* iff ${\text {reach}}(M)>0$.

The following generalizes [7, Lemma], which states that compact $C^2$ submanifolds have positive reach.

### **Proposition 6**

*Let* *M* *be a* $C^{1,1}$ *submanifold*. *Then* ${\text {reach}}(M)\le \inf \{\vartheta (\xi ,v):(\xi ,v)\in \nu _1(M)\}$, *with equality if* *M* *is a closed subset of* ${{\mathbb {R}}}^d$.

*In particular*, *if* *M* *is compact*, *then* ${\text {reach}}(M)=\min \{\vartheta (\xi ,v):(\xi ,v)\in \nu _1(M)\}>0$.

### *Proof*

If ${\text {reach}}(M)=0$, then trivially ${\text {reach}}(M)\le \inf \{\vartheta (\xi ,v):(\xi ,v)\in \nu _1(M)\}$. If ${\text {reach}}(M)>0$, let $\varepsilon \in \big (0,{\text {reach}}(M)\big )$. Then $M^\varepsilon$ is open and $M^\varepsilon \subseteq {\text {unpp}}(M)$. Thus $M^\varepsilon \subseteq {\mathscr {E}}(M)$. So for every $(\xi ,v)\in \nu _1(M)$ and every $r\in (0,\varepsilon )$ we have $\xi +rv\in {\mathscr {E}}(M)$ and thus $r<\vartheta (\xi ,v)$, so $\varepsilon \le \vartheta (\xi ,v)$. From this it follows that ${\text {reach}}(M)\le \vartheta (\xi ,v)$ for all $(\xi ,v)\in \nu _1(M)$.

Suppose now that *M* is a closed subset of ${{\mathbb {R}}}^d$. If $\inf \{\vartheta (\xi ,v):(\xi ,v)\in \nu _1(M)\}=0$ there is nothing to show. Otherwise let $\varepsilon \in (0,\infty )$ with $\varepsilon <\inf \{\vartheta (\xi ,v):(\xi ,v)\in \nu _1(M)\}$ and let $x\in M^\varepsilon \setminus M$. Let $\xi \in M$ be a nearest point to *x* on *M*. Then $(\xi ,x-\xi )\in \nu (M)$ by Remark 4. We have $\vartheta (\xi ,\tfrac{x-\xi }{\Vert x-\xi \Vert })>\varepsilon$, so $\xi +r \tfrac{x-\xi }{\Vert x-\xi \Vert }\in {\mathscr {E}}(M)$ for all $r\in (0,\varepsilon )$. In particular, $x\in {\mathscr {E}}(M)$ and $\xi =p(x)$. So $M^\varepsilon \subseteq {\text {unpp}}(M)$, and therefore ${\text {reach}}(M)\ge \varepsilon$.

Now assume *M* is compact. Then also $\nu _1(M)$ is compact since it is homeomorphic to the product of compact spaces. By Theorem A, $\vartheta$ is lower semi-continuous on $\nu _1(M)$, and therefore attains its minimum in some point $(\xi _0,v_0)\in \nu _1(M)$. By Theorem 1, $\vartheta (\xi _0,v_0)>0$. $\square$

### *Remark 7*

Let $M\subseteq {{\mathbb {R}}}^d$. If *M* has positive reach, then *M* is closed: suppose *M* were not closed. Let $z \in {\bar{M}} \setminus M$. Then *z* has no nearest point on *M*, and $z\in M^\varepsilon$ for all $\varepsilon \in (0,\infty )$. Thus, $M^\varepsilon \notin {\text {unpp}}(M)$ for every $\varepsilon \in (0,\infty )$, so that ${\text {reach}}(M)=0$.

## Derivatives of *p* and $\delta _M$

A proof of the following theorem can be found in [5]. We give a different proof here, which makes it an immediate consequence of Lemmas 4 and 6.

### **Theorem 2**

*Let* *M* *be a* $C^k$-*submanifold of* ${{\mathbb {R}}}^d$ *with* $k\ge 1$. *Then* *p* *is* $C^{k-1}$ *on* ${\mathscr {E}}(M)$ *and* $\delta _M$ *is* $C^k$ *on* ${\mathscr {E}}(M)\setminus M$.

### *Proof*

If $k=1$ then the claim is only that *p* is continuous and $\delta _M$ is differentiable. But continuity of *p* is the content of Proposition 1, differentiability of $\delta _M$ is settled by Foote [7, Theorem 2].

Consider now the case $k\ge 2$. We show that *p* is differentiable. We have already shown in Lemma 6 that $F^*:\nu ^*(M)\rightarrow {\mathscr {E}}(M)$ is a diffeomorphism, and by Lemma 4, $p(F^*(\xi ,v))=\xi$ for all $(\xi ,v)\in \nu ^*(M)$. We have $(F^*)^{-1}(x)=(p(x),x-p(x))$ for all $x\in {\mathscr {E}}(M)$, so that

$$\begin{aligned} p(x)=F^*(p(x),0)=F^*({\tilde{p}}(p(x),x-p(x)))=F^*(\tilde{p}((F^*)^{-1}(x))) \end{aligned}$$

for all $x\in {\mathscr {E}}(M)$, where ${\tilde{p}}:\nu (M)\rightarrow \nu (M)$ is the projection onto $M\times \{0\}\subseteq \nu (M)$.

We obtain $p=F^*\circ {\tilde{p}}\circ (F^*)^{-1}$. The function $F^*$ is $C^{k-1}$ and so is therefore also $(F^*)^{-1}$. The projection ${\tilde{p}}$ is clearly $C^{k-1}$, and so is *p*.

For the regularity of $\delta _M$ we use the argument given in [7], which we repeat here for convenience of the reader: $\delta _M(x)=\Vert x-p(x)\Vert$ for all $x\in {\mathscr {E}}(M)$. Then for $x\in {\mathscr {E}}(M)\setminus M$,

$$\begin{aligned} (D(\delta _M^2))(x) v=2(x-p(x))^\top (v-(Dp(x)) v). \end{aligned}$$

Since $p:{\mathscr {E}}(M)\rightarrow M$ and thus *Dp* is a mapping between the tangent bundles, i.e., $Dp:T({\mathscr {E}}(M))\rightarrow T(M)$, $Dp(x)v\in T_{p(x)}(M)$, so that $(x-p(x))^\top Dp(x) v=0$. Hence $(D \delta _M^2)(x)=2(x-p(x))^\top$, which is $C^{k-1}$, since this holds for *p*. Thus $\delta _M$ is $C^k$. $\square$

### *Remark 8*

In contrast to *p*, the distance function is defined on the whole of ${{\mathbb {R}}}^d$. Moreover, $\delta _M$ is continuous on ${{\mathbb {R}}}^d$. However, it is easy to find examples of $C^\infty$-submanifolds so that $\delta _M$ is not differentiable on ${{\mathbb {R}}}^d\setminus M$, for example, if $M=\{(x,y)\in {{\mathbb {R}}}^2:y=x^2 \}$, then $\delta _M$ is not differentiable in the points $\{(0,y):y> 1/2\}$.

Dudek and Holly [5, Lemma 4′.1] compute the (Frechet-)derivative of *p* . In the following theorem we give a slightly different form which is more suitable for showing the subsequent Corollaries 3 and 4. The proof uses a method different from that in [5].

### **Theorem C**

*Let* *M* *be a* $C^2$ *submanifold*. *For every* $x\in {\mathscr {E}}(M)\setminus M$ *the differential of* *p* *in* *x* *is given by*

$$\begin{aligned} Dp(x)&=\Big ({\text {id}}_{T_{p(x)}(M)}-\Vert x-p(x)\Vert L_{p(x),v}\Big )^{-1}P_{T_{p(x)}(M)}, \end{aligned}$$

*where* $v=\Vert x-p(x)\Vert ^{-1}(x-p(x))$ *and* $L_{p(x),v}$ *is the shape operator in direction* *v* *at* *p*(*x*). *For every* $x\in M$ *the differential of* *p* *in* *x* *is* $Dp(x) =P_{T_{p(x)}(M)}.$

We see that, in general, *Dp*(*x*) may explode as *x* approaches the boundary of ${\mathscr {E}}(M)$, even for *M* that are well behaved, like a circle in the plane. The next two results state that the higher-order differentials of *p* on $M^\varepsilon \setminus M$ with $\varepsilon <{\text {reach}}(M)$ are bounded, provided the higher-reivatives of normal vectors are bounded.

### **Corollary 3**

*Let* $M\subseteq {{\mathbb {R}}}^d$ *be a* $C^k$ *submanifold with* $k\ge 2$, *let* ${\text {reach}}(M)>0$ *and* $\varepsilon \in (0,{\text {reach}}(M))$. *Suppose there exist a constant* $K\in (0,\infty )$ *and a family of* $C^{k-1}$ *moving frames* $(V_i,n_{i,m+1},\ldots ,n_{i,d})_{i\in I}$ *such that* $M=\bigcup _{i\in I} V_i$ *and* $\Vert D^jn_{i,\ell }\Vert$ *is bounded by* *K* *for every* $i\in I$, $\ell \in \{m+1,\ldots ,d\}$ *and* $j\in \{2,\ldots ,k-1\}$. *Then* $D^jp$ *is bounded on* $M^{\varepsilon }$, *for every* $j\in \{2,\ldots ,k-1\}$.

### *Proof*

Let $x\in M^\varepsilon$, i.e., $\Vert x-p(x)\Vert <\varepsilon$. Let $\lambda _1,\ldots ,\lambda _m$ be the eigenvalues of $L_{\xi ,v}$. Since $L_{\xi ,v}$ is self-adjoint by Theorem 4, $\Vert L_{\xi ,v}\Vert =\max (|\lambda _1|,\ldots ,|\lambda _m|)$, and there is no loss of generality in assuming $\Vert L_{\xi ,v}\Vert =|\lambda _1|$.

If $\lambda _1\ge 0$, then by Theorem 4, $\Vert L_{\xi ,v}\Vert =\varrho (\xi ,v)^{-1}$. We have $\Vert x-p(x)\Vert<\varepsilon <\varepsilon _0\le \vartheta (\xi ,v)\le \varrho (\xi ,v)$, by $x\in M^\varepsilon$, Theorem 6, and Lemma 5. Therefore,

$$\begin{aligned} \big \Vert \Vert x-p(x)\Vert L_{p(x),v}\big \Vert = \Vert x-p(x)\Vert \left\| L_{p(x),v}\right\|<\varepsilon \varrho (\xi ,v)^{-1} \le \frac{\varepsilon }{\varepsilon _0}<1. \end{aligned}$$

If $\lambda _1<0$, then $\Vert L_{\xi ,v}\Vert =\Vert L_{\xi ,-v}\Vert =-\lambda _1=\varrho (\xi ,-v)$. From this we get $\Vert x-p(x)\Vert<\varepsilon <\varepsilon _0\le \vartheta (\xi ,-v)\le \varrho (\xi ,-v)$, and thus again

$$\begin{aligned} \big \Vert \Vert x-p(x)\Vert L_{p(x),v}\big \Vert = \Vert x-p(x)\Vert \left\| L_{p(x),-v}\right\|<\varepsilon \varrho (\xi ,-v)^{-1} \le \frac{\varepsilon }{\varepsilon _0}<1. \end{aligned}$$

Therefore ${\text {id}}_{T_{p(x)}(M)}-\Vert x-p(x)\Vert L_{p(x),v}$ with $v=\tfrac{x-p(x)}{\Vert x-p(x)\Vert }$ is invertible, and

$$\begin{aligned} \Big \Vert \big ({\text {id}}_{T_{p(x)}(M)}-\Vert x-p(x)\Vert L_{p(x),v}\big )^{-1}\Big \Vert&=\Big \Vert \sum _{\ell =0}^\infty \big (\Vert x-p(x)\Vert L_{p(x),v})^\ell \Big \Vert \\&\le \sum _{\ell =0}^\infty \big \Vert \Vert x-p(x)\Vert L_{p(x),v}\big \Vert ^\ell \\&\le \sum _{\ell =0}^\infty \left( \frac{\varepsilon }{\varepsilon _0}\right) ^\ell <\big (1-\frac{\varepsilon }{\varepsilon _0}\big )^{-1}. \end{aligned}$$

Let now $(V,n_{m+1},\ldots ,n_d)$ be a moving frame with $x\in V$ and differentials bounded by *K*. Write down equation (7) and write *n* for the matrix $(n_{m+1},\ldots ,n_d)$:

$$\begin{aligned} {\text {id}}_{{{\mathbb {R}}}^d}-n(p(x))n(p(x))^\top&=J(x)Dp(x), \end{aligned}$$

where $J(x):=\Big ({\text {id}}_{T_{p(x)}(M)}+\sum _j\big \langle x-p(x), n_j(p(x))\big \rangle (D n_j)(p(x))\Big )$. From this and the fact that *J*(*x*) is invertible with uniformly bounded differential, we get

3 $$\begin{aligned} Dp(x)= J(x)^{-1}\big ({\text {id}}_{{{\mathbb {R}}}^d}-n(p(x))n(p(x))^\top \big ) \end{aligned}$$

and thus *Dp* is uniformly bounded. Differentiating the right-hand side of (3) involves sums of products of *n*, *Dn*, $D^2n$, *p*, *Dp*, their rows, columns and transposes, and $J(x)^{-1}$, which are all bounded. From this we get boundedness of $D^2p$. By induction we get boundedness of $D^jp$, $j\in \{1,\ldots ,k-1\}$ from boundedness of $D^jn$, $j\in \{1,\ldots ,k-1\}$. $\square$

The situation simplifies if *M* is a hypersurface, i.e., a $(d-1)$-dimensional submanifold of ${{\mathbb {R}}}^d$. Then on every connected open subset $V\subseteq M$ there exist at most two $C^1$ functions *n* with the property that (*V*, *n*) is a unit normal field, and those functions differ only by their sign. If *M* is not orientable, then (per definition) it is not possible to find a unit normal vector (*V*, *n*) with $V=M$. Nevertheless, every (*V*, *n*) is automatically $C^{k-1}$ and $\sup _{\xi \in M}\Vert D^jn(\xi )\Vert$ does not depend on the choice of a particular unit normal vector. With this, the following corollary is an immediate consequence of Corollary 3.

### **Corollary 4**

*Let* $M\subseteq {{\mathbb {R}}}^d$ *be a* $C^k$ *hypersurface with* $k\ge 2$. *Moreover*, *let* ${\text {reach}}(M)>0$ *and* $\varepsilon \in (0,{\text {reach}}(M))$. *If there exists* $K\in (0,\infty )$ *such that* $\sup _{\xi \in M}\Vert D^jn\Vert \le K$ *for every* $j\in \{2,\ldots ,k-1\}$, *then* $D^jp$ *is bounded on* $M^{\varepsilon }$ *for every* $j\in \{2,\ldots ,k-1\}$.

## The converse to Theorem 1

In their article [5] from 1994, Dudek and Holly prove that each point of a $C^{1,1}$ submanifold of ${{\mathbb {R}}}^d$ (see Definition 3) with dimension different from *d* possesses a neighborhood in the ambient space ${{\mathbb {R}}}^d$ which has the ${\text {unpp}}$. This was the assertion of Theorem 1, or [5, Theorem 3.8] in the original paper. In Theorem B in this section we show the converse to the theorem, starting from topological submanifolds: if a topological submanifold *M* of ${{\mathbb {R}}}^d$ with dimension $m\ne d$ is such that each point $\xi$ of *M* has an ${{\mathbb {R}}}^d$-neighborhood $U(\xi )$ such that $U(\xi )\subseteq {\text {unpp}}(M)$, then *M* is $C^{1,1}$.

Theorem B will be formulated and proven after two essential lemmas below. The proof’s core is Lemma 8, which allows the construction of normal and tangent spaces to *M* by merely using the property that each point of *M* has a neighborhood admitting unique projections onto *M*. The proof of Lemma 8 relies on an iterative application of the Borsuk–Ulam theorem. We shall also use the following lemma.

### **Lemma 7**

*Let* *M* *be a subset of* ${{\mathbb {R}}}^d$ *and* $U\subseteq {\text {unpp}}(M)$. *If* *U* *is convex*, *then for every* $\xi \in M$ *the set* $U\cap p^{-1}(\xi )$ *is convex*.

### *Proof*

Let $x_1,x_2\in U$ with $p(x_1)=p(x_2)=\xi$, and let $x_3\in {]x_1,x_2[}$. Then it is not hard to check that $B_{\Vert x_3-\xi \Vert }(x_3)\subseteq B_{\Vert x_1-\xi \Vert }(x_1)\cup B_{\Vert x_2-\xi \Vert }(x_2)$. Since $\big (B_{\Vert x_1-\xi \Vert }(x_1)\cup B_{\Vert x_2-\xi \Vert }(x_2)\big )\cap M=\emptyset$, $\big (\bar{B}_{\Vert x_1-\xi \Vert }(x_1)\cup {\bar{B}}_{\Vert x_2-\xi \Vert }(x_2)\big )\cap M=\{\xi \}$ we have $B_{\Vert x_3-\xi \Vert }(x_3)\cap M=\emptyset$ and $\bar{B}_{\Vert x_3-\xi \Vert }(x_3)\cap M\subseteq \{\xi \}$. On the other hand clearly $\xi \in {\bar{B}}_{\Vert x_3-\xi \Vert }(x_3)\cap M$, and therefore $\{\xi \}= {\bar{B}}_{\Vert x_3-\xi \Vert }(x_3)\cap M$. $\square$

We remind the reader that for a set $A\subseteq {{\mathbb {R}}}^d$ and $r\in (0,\infty )$ we denote $A^r=\{x\in {{\mathbb {R}}}^d:d(x,A)<r\}$. For example, if *H* is a 2-dimensional subspace of ${{\mathbb {R}}}^3$ and $S=\bar{B}_1(0)\setminus B_1(0)$, $0<r_1\le r_2$, then $A=\big ((r_2 S)\cap H\big )^{r_1}$ is the interior of a filled torus, the case $r_1=r_2$ giving a “horn torus”. Note that $A\cap H^\perp =\emptyset$, even in the latter case.

### **Lemma 8**

*Let* *M* *be an* *m*-*dimensional topological submanifold of* ${{\mathbb {R}}}^d$ *with the property that for every* $\eta \in M$ *there exists* $U\subseteq {\text {unpp}}(M)$ *with* *U* *open and* $\eta \in U$. *Then for every* $\eta \in M$ *there exists* $r\in (0,\infty )$ *such that for every* $\xi \in M\cap B_r(\eta )$ *there exists a* $(d-m)$-*dimensional subspace* $N_\xi$ *of* ${{\mathbb {R}}}^d$ *with*

4 $$\begin{aligned} \Big (\xi +\big ((r S)\cap N_\xi \big )\Big )^r\cap M=\emptyset \quad {\mathrm{and}}\quad \overline{\Big (\xi +\big ((r S)\cap N_\xi \big )\Big )^r}\cap M=\{\xi \}, \end{aligned}$$

*where* *S* *denotes the* $(d-1)$-*dimensional unit sphere*.

### *Proof*

There exist open sets $V,W\subseteq {{\mathbb {R}}}^d$ with $0\in V$, $\eta \in W$ and a continuous map $\varPsi :V\rightarrow W$ with $\varPsi (0)=\eta$ and $M\cap W=\{\varPsi (y):y\in V,\,y_{m+1}=\cdots =y_d=0\}$. There exists $r\in (0,\infty )$ such that $B_{3r}(\eta )\subseteq U\cap W$. Let $\xi \in M\cap B_r(\eta )$. Denote by $\pi _{d-1}:{{\mathbb {R}}}^d\rightarrow {{\mathbb {R}}}^{d-1}$ the projection defined by $\pi _{d-1}(y_1,\ldots ,y_d):=(y_1,\ldots ,y_{d-1})$. By Proposition 1, and since $\xi +r S\subseteq B_{3r}(\eta )\subseteq U\subseteq {\text {unpp}}(M)$, $f_1:S \rightarrow {{\mathbb {R}}}^{d-1}$, $f_1(v):=\pi _{d-1}\big (\varPsi ^{-1}(p(\xi +rv))\big )$ is continuous. By the Borsuk–Ulam theorem there exists $n_d\in S$ with $f_1(n_d)=f_1(-n_d)$, yielding $p(\xi +rn_d)=p(\xi -rn_d)=:\zeta$. Obviously, $\Vert \xi +rn_d-\zeta \Vert \le r$ and $\Vert \xi -rn_d-\zeta \Vert \le r$ and $\Vert \xi +rn_d-(\xi -rn_d)\Vert =2r$. Thus, by the triangle inequality $\Vert \xi +rn_d-\zeta \Vert = r$ and $\Vert \xi -rn_d-\zeta \Vert = r$ and therefore $\zeta =\xi$. Let $N^{(1)}$ be the hyperplane $\{v\in {{\mathbb {R}}}^d:\langle v,n_d\rangle =0\}=n_d^\perp$.

Now let $\pi _{d-2}:{{\mathbb {R}}}^d\rightarrow {{\mathbb {R}}}^{d-2}$ be the projection defined by $\pi _{d-2}(y_1,\ldots ,y_d):=(y_1,\ldots ,y_{d-2})$, and let $f_2:S\cap N^{(1)} \rightarrow {{\mathbb {R}}}^{d-2}$, $f_2(v):=\pi _{d-2}\big (\varPsi ^{-1}(p(\xi +rv))\big )$. Applying the Borsuk–Ulam theorem again yields $n_{d-1}\in S\cap N^{(1)}$ with $p(\xi +rn_{d-1})=p(\xi -rn_{d-1})=\xi$, as before. Let $N^{(2)}$ be the $d-2$ dimensional space $N^{(2)}=\{v\in {{\mathbb {R}}}^d:\langle v,n_{d-1}\rangle =0\text { and }\langle v,n_d\rangle =0\}=n_{d-1}^\perp \cap n_d^\perp$.

By iterating this procedure we get $d-m$ orthonormal vectors $n_{m+1},\ldots ,n_d$ for which $p(\xi +rn_j)=p(\xi -rn_j)=\xi$, $j=m+1,\ldots ,d$, and $d-m$ subspaces $N_\xi :=N^{(d-m)}\subseteq \cdots \subseteq N^{(1)}$. Note that $N_\xi$ is the linear space spanned by $n_{m+1},\ldots ,n_d$.

Let

$$\begin{aligned} K:=r{\text {conv}}\{n_{m+1},\ldots ,n_d,-n_{m+1},\ldots ,-n_d\}, \end{aligned}$$

where ${\text {conv}}(A)$ denotes the convex hull of a set $A\subseteq {{\mathbb {R}}}^d$. By construction of the $n_j$’s and by Lemma 7, we have $p(\xi +K)=\{\xi \}$. Using Lemma 3 we get $p\Big (\xi +\big ((rS)\cap N_\xi \big )\Big )=\{\xi \}$. From this the assertion follows. $\square$

If $t\in {{\mathbb {R}}}^d\setminus \{0\}$ is a vector and $T\subseteq {{\mathbb {R}}}^d$ is a non-trivial linear subspace, then ${\sphericalangle }(t,T):=\min \{{\sphericalangle }(t,t_2):t_2\in T\setminus \{0\}\}$.

If $T_1,T_2\subseteq {{\mathbb {R}}}^d$ are two non-trivial linear subspaces, we define

$$\begin{aligned} {\sphericalangle }(T_1,T_2):=\max \big \{\min \{{\sphericalangle }(t_1,t_2):t_2\in T_2\setminus \{0\}\}:t_1\in T_1\setminus \{0\}\big \}. \end{aligned}$$

Note that $d_H(T_1,T_2):=2\arcsin ({\sphericalangle }(T_1,T_2)/2)$ for $T_1,T_2\in G(m,{{\mathbb {R}}}^d)$.

### **Theorem B**

*Let* *M* *be an* *m*-*dimensional topological submanifold of* ${{\mathbb {R}}}^d$ *with* $M\subseteq {\mathscr {E}}(M)$. *Then* *M* *is* $C^{1,1}$.

### *Proof*

Step 1 We show that *M* is locally the graph of a function $\varPhi$ over an *m*-dimensional subspace of ${{\mathbb {R}}}^d$.

Let $\eta \in M$. By Lemma 8 there exists $r\in (0,\infty )$ such that for every $\xi \in M\cap B_r(\eta )$ there exists a $(d-m)$-dimensional subspace $N_\xi$ of ${{\mathbb {R}}}^d$ and such that (4) is satisfied. Moreover, *r* can by chosen so that $M_1:=M\cap B_r(\eta )$ is homeomorphic to an open subset of ${{\mathbb {R}}}^m$. For every $\xi \in M$ with $\Vert \xi -\eta \Vert <r$ write $T_\xi :=N_\xi ^\perp$, where $N_\xi$ is the linear space constructed in Lemma 8. Consider the map $f:M_1\rightarrow T_\eta$, $f(\xi )=P_{T_\eta }(\xi -\eta )$. The map *f* is injective: suppose to the contrary that there exist $\xi ,\zeta \in M_1$ with $P_{T_\eta }(\xi -\eta )=P_{T_\eta }(\zeta -\eta )$, so ${\sphericalangle }(\zeta -\xi ,T_\eta )=\frac{\pi }{2}$. By (4) and $\Vert \xi -\eta \Vert <r$ we have ${\sphericalangle }(\xi -\eta ,T_\eta )<\frac{\pi }{6}$, and similarly ${\sphericalangle }(\eta -\xi ,T_\xi )<\frac{\pi }{6}$, so that ${\sphericalangle }(T_\xi ,T_\eta )<\frac{\pi }{3}$. We also have ${\sphericalangle }(\zeta -\xi ,T_\xi )<\frac{\pi }{6}$. But ${\sphericalangle }(\zeta -\xi ,T_\eta )\le {\sphericalangle }(\zeta -\xi ,T_\xi )+{\sphericalangle }(T_\xi ,T_\eta )<\frac{\pi }{6}+\frac{\pi }{3}<\frac{\pi }{2}$, a contradiction.

The set $V:=\{P_{T_\eta }(\xi -\eta ):\xi \in M_1\}$ is open in $T_\eta$ by Brouwer’s invariance of domain theorem. Thus, the map $\varPhi :V \rightarrow N_\eta$, $\varPhi (t)=f^{-1}(t)-\eta -t$ provides us with a parametrization of $M_1$ via $t\mapsto \eta +t+\varPhi (t)$. We see that $M_1$ is the graph of a function.

Step 2 We show that $\varPhi$ is differentiable.

Let $t\in V$. Then $\xi :=\eta +t+\varPhi (t)\in M_1$ and ${\sphericalangle }(T_\xi ,T_\eta )<\frac{\pi }{3}$ such that $T_\xi \cap (T_\eta ^\perp )=\{0\}$. Thus, there exists a linear mapping $A_t:T_\eta \rightarrow T_\eta ^\perp$ with $T_\xi =\{h+A_t h:h\in T_\eta \}$. Since ${\sphericalangle }(T_\eta ,T_\xi )<\frac{\pi }{3}$ and because of (4) it holds $-\frac{8}{r}\Vert h\Vert ^2\le \Vert \varPhi (t+h)-\varPhi (t)-A_t h\Vert \le \frac{8}{r}\Vert h\Vert ^2$ for all *h* with sufficiently small norm. Thus, $\varPhi$ is differentiable in *t*, with differential $A_t$. In particular, $T_\xi (M)=T_\xi$ for every $\xi \in M$.

Step 3 We show that the differential of $\varPhi$ is Lipschitz so that, in particular, *M* is $C^1$.

So far we have succeeded in showing that for all $t\in V$ and all $h\in T_\eta$ satisfying $t+h\in V$ it holds

$$\begin{aligned} \varPhi (t+h)=\varPhi (t)+A_t h +\kappa (t,h) \end{aligned}$$

for some remainder function $\kappa$ satisfying $\Vert \kappa (t,h)\Vert <\frac{8}{r}\Vert h\Vert ^2$.

Now fix $t\in V$ and let *a* be such that $B_a(t)\cap T_\eta \subseteq V$. Let $h,k\in T_\eta$ with $0<\Vert h\Vert =\Vert k\Vert <\frac{a}{2}$. Then

$$\begin{aligned} \varPhi (t+k)&=\varPhi (t+h)+ A_{t+h}(k-h) +\kappa (t+h,k-h)\\ \varPhi (t-k)&=\varPhi (t+h)+A_{t+h}(-k-h) +\kappa (t+h,-k-h)\\ \varPhi (t+k)&=\varPhi (t)+ A_tk +\kappa (t,k)\\ \varPhi (t-k)&=\varPhi (t)+ A_t(-k) +\kappa (t,-k). \end{aligned}$$

We add the first and fourth equation and subtract the second and third to get

$$\begin{aligned} 0=2 (A_{t+h}-A_t)k+\kappa (t+h,k-h)-\kappa (t+h,-k-h)-\kappa (t,k)+\kappa (t,-k) \end{aligned}$$

and thus

$$\begin{aligned} 2 \Vert (A_{t+h}-A_t)k\Vert&\le \frac{8}{r}(\Vert k-h\Vert ^2+\Vert -k-h\Vert ^2+\Vert k\Vert ^2+\Vert -k\Vert ^2)\\&= \frac{8}{r}\big (2\Vert h\Vert ^2+4\Vert k\Vert ^2\big )=\frac{48}{r}\Vert h\Vert ^2, \end{aligned}$$

since we assumed $\Vert k\Vert =\Vert h\Vert$. Therefore,

$$\begin{aligned} \Vert k\Vert ^{-1}\Vert (A_{t+h}-A_t)k\Vert&\le \frac{24}{r}\Vert k\Vert ^{-1}\Vert h\Vert ^2=\frac{24}{r}\Vert h\Vert , \end{aligned}$$

and, because *k* was arbitrary with $\Vert k\Vert =\Vert h\Vert$, it follows that $\Vert A_{t+h}-A_t\Vert \le \frac{24}{r}\Vert h\Vert$, which means that the mapping $t\mapsto A_t$ is Lipschitz.

Step 4 We show that *M* is $C^{1,1}$. For this it suffices to show that $\xi \mapsto T_\xi$ is Lipschitz on $M\cap B_\frac{r}{4}(\eta )$. Let now $\xi ,\zeta \in M\cap B_\frac{r}{4}(\eta )$, thus $\zeta \in M\cap B_{\frac{r}{2}}(\xi )$. Note that since $\xi \in M\cap B_\frac{r}{4}(\eta )$, clearly $M\cap B_{\frac{r}{2}}(\xi )\subseteq M_1$ and therefore Step 1–Step 3 can be performed for $\xi , M\cap B_{\frac{r}{2}}(\xi )$ in position of $\eta , M_1$, yielding also the same Lipschitz constant in Step 3.

In particular, as done for $\eta$ in Step 1, $M\cap B_\frac{r}{2}(\xi )$ can be represented as a graph over $T_\xi$: there exists ${\bar{\varPhi }}:T_\xi \cap B_\frac{r}{2}(0)\rightarrow N_\xi$ such that $M\cap \{\xi +(T_\xi \cap B_\frac{r}{2}(0))+(N_\xi \cap B_r(0))\} =\{\xi +t+{\bar{\varPhi }}(t):t\in T_\xi \cap B_\frac{r}{2}(0)\}$ and $\bar{\varPhi }(0)=0$.

Hence, $\zeta =\xi +t+{\bar{\varPhi }}(t)$ for some $t\in T_\xi$, and $T_\zeta =\{t_1+D{\bar{\varPhi }}(t)t_1:t_1\in T_\xi \}$. Now let $s\in T_\xi$ with $\Vert s\Vert =1$ and such that ${\sphericalangle }(T_\xi ,T_\zeta )=\arctan (\Vert D{\bar{\varPhi }}(t)s\Vert )$ and hence ${\sphericalangle }(T_\xi ,T_\zeta )\le \arctan (\Vert D{\bar{\varPhi }}(t)\Vert )$. We use the estimate $2 \arcsin \left( \tfrac{1}{2}\arctan (x)\right) \le x$ for $x\in [0,\infty )$ (which can be shown by proving that *g* defined by $g(x):=x-2 \arcsin \left( \tfrac{1}{2}\arctan (x)\right)$ satisfies $g(0)=0$ and $g'>0$), the Lipschitz continuity of ${\bar{\varPhi }}$ from Step 3 and Pythagoras’ theorem to compute the estimate

$$\begin{aligned} d_H(T_\xi ,T_\zeta )&=2 \arcsin \left( \tfrac{1}{2}\arctan (\Vert D{\bar{\varPhi }}(t)\Vert )\right) \le \Vert D{\bar{\varPhi }}(t)\Vert \\&\le \tfrac{24}{r}\Vert t\Vert \le \tfrac{24}{r}\Vert t+{\bar{\varPhi }}(t)\Vert = \tfrac{24}{r}\Vert \xi -\zeta \Vert . \end{aligned}$$

$$\square $$

### *Remark 9*

[21, Theorem 1] generalizes Blaschke’s Rolling Theorem for the boundary of a compact and path-connected subset $P\subseteq {{\mathbb {R}}}^d$. In particular, the theorem there states that there exists $r_0>0$ such that a ball of radius *r* rolls freely inside *P* and $\overline{P^c}$ for all $0\le r\le r_0$ iff $\partial P$ is a $C^{1,1}$ hypersurface.

For hypersurfaces this free-rolling condition is equivalent to ${\text {reach}}(\partial P)\ge r_0$, using the notation of the present article. However, the methods used there cannot be used to prove Theorem B, and also there is no obvious way to generalize the whole setup in [21] to codimensions other than 1.

## The topological skeleton

Here, we highlight the relation between ${\mathscr {E}}(M)$ and the topological skeleton (a.k.a. medial axis) of $M^c$.

### **Definition 9**

Let $A\subseteq {{\mathbb {R}}}^d$ be a subset.

1. A ball $B_r(x)$ with $r\in (0,\infty )$ is called *maximal in A*, iff
  1. $$B_r(x)\subseteq A$$ ;
  2. for all $x_1\in {{\mathbb {R}}}^d, r_1\in (0,\infty ):B_r(x)\subseteq B_{r_1}(x_1)\subseteq A$, then $r_1=r,x_1=x$.
2. Define the *topological skeleton* by
  $$\begin{aligned} {\mathscr {S}}(A):=\{x\in {{\mathbb {R}}}^d:\exists r\in (0,\infty ) :B_r(x) \text { is maximal in }A\} \end{aligned}$$

### *Remark 10*

Note that a ball $B_r(x)$ is maximal in *A* iff $B_r(x)$ is maximal in $A^\circ$. Therefore, ${\mathscr {S}}(A)={\mathscr {S}}(A^\circ )$.

Note further that if *x* has at least 2 nearest points on $A^c$, then $x\in {\mathscr {S}}(A)$.

The following is an adaptation of the well-known medial axis transform, which allows reconstruction of an open subset of ${{\mathbb {R}}}^d$ from its topological skeleton. In our version the complement of a closed set (for example a closed manifold) is recovered from its skeleton.

### **Proposition 7**

(Medial axis transform, recovery from skeleton) *Let* $M\subseteq {{\mathbb {R}}}^d$ *be a closed subset*. *Let* ${\mathscr {H}}(M)$ *be the set of all closed half-spaces containing* *M*. *Then*

$$\begin{aligned} M^c=\Bigg (\bigcup _{H\in {\mathscr {H}}(M)}H^c\Bigg ) \cup \bigcup \big \{B_r(x):x\in {\mathscr {S}}(M^c)\text { and } r=d(x,M)\big \}. \end{aligned}$$

### *Proof*

Let $${\mathscr {B}}=\bigcup \big \{B_r(x):x\in {\mathscr {S}}(M^c)\text { and } r=d(x,M)\big \}$$.

The inclusion $\Big (\bigcup _{H\in {\mathscr {H}}(M)}H^c\Big ) \cup {\mathscr {B}}\subseteq M^c$ is obvious.

We show $M^c\subseteq \Big (\bigcup _{H\in {\mathscr {H}}(M)}H^c\Big ) \cup {\mathscr {B}}$. Note that $\bigcup _{H\in {\mathscr {H}}(M)}H^c$ equals the complement of the convex hull ${\text {conv}}(M)$ of *M*. Now let $x\in M^c\setminus {\text {conv}}(M)^c$. Since *M* is closed, *x* has nearest points on *M*. Consider first the case that *x* has 2 or more distinct nearest points. Then $B_{d(x,M)}(x)$ is maximal in $M^c$. Thus, $x\in {\mathscr {S}}(M^c)$ and therefore $x\in B_{d(x,M)}(x)\subseteq {\mathscr {B}}$.

Next, consider the case that *x* has a unique nearest point $\zeta$ on *M*, and let $\alpha =\sup \{a\in [1,\infty ):B_{a\Vert x-\zeta \Vert }\big (\zeta +a(x-\zeta )\big )\subseteq M^c\}$. Since the set over which the supremum is taken contains 1, we see that $\alpha \in [1,\infty ]$.

If $\alpha =\infty$ then $B_{a\Vert x-\zeta \Vert }\big (\zeta +a(x-\zeta )\big )\subseteq M^c$ for arbitrarily large *a*, so $x\in H^c$ for the closed half-space $H=\{z\in {{\mathbb {R}}}^d:\langle z-\zeta ,x-\zeta \rangle \le 0\}$. Thus $x\in {\text {conv}}(M)^c$, which was excluded.

If $\alpha \in [1,\infty )$ it is easy to show that the ball $B_{\alpha \Vert x-\zeta \Vert }\big (\zeta +\alpha (x-\zeta )\big )$ is maximal. So $\zeta +\alpha (x-\zeta )\in {\mathscr {S}}(M^c)$ and $x\in B_{\Vert x-\zeta \Vert }(x)\subseteq B_{\alpha \Vert x-\zeta \Vert }\big (\zeta +\alpha (x-\zeta )\big )\subseteq {\mathscr {B}}$. $\square$

The next result describes the relation between ${\mathscr {E}}(M)$ and the skeleton of $M^c$.

### **Proposition 8**

*Let* $$M\subseteq {{\mathbb {R}}}^d$$. *Then*

$$\begin{aligned} {\mathscr {E}}(M)^c =\overline{{\mathscr {S}}\big ( M^c\big )} \cup \overline{{\mathscr {F}}(M)} =\overline{{\mathscr {S}}\Big (\big ({{\overline{M}}}\big )^c\Big )} \cup \overline{{\mathscr {F}}(M)}, \end{aligned}$$

*where* ${\mathscr {F}}(M)$ *is the set of points* $x\in {{\mathbb {R}}}^d$ *for which there is no nearest point to* *x* *in* *M*.

### *Proof*

Since $\big ({\overline{M}})^c=\big (M^c\big )^\circ$, ${\mathscr {S}}\Big (\big ({\overline{M}}\big )^c\Big )={\mathscr {S}}(M^c)$, it is enough to prove the first equality.

Step 1 We show ${\mathscr {E}}(M)^c\subseteq \overline{{\mathscr {S}}(M^c)} \cup \overline{{\mathscr {F}}(M)}$.

Let $x\in {\mathscr {E}}(M)^c$. W.l.o.g. there exists a sequence $(x_n)_{n\in {{\mathbb {N}}}}$ converging to *x* such that either all $x_n$ have no nearest point on *M* or all $x_n$ have multiple nearest points on *M*.

In the first case, all $x_n$ are in ${\mathscr {F}}(M)$ by definition. Therefore $x\in \overline{{\mathscr {F}}(M)}$. In the second case, all $x_n$ have multiple nearest points on *M*. Thus $x_n$ is the center of a maximal ball in $M^c$ and therefore $x_n$ in ${\mathscr {S}}(M^c)$. Hence $x\in \overline{{\mathscr {S}}(M^c)}$.

Step 2 We show $\overline{{\mathscr {S}}( M^c)} \cup \overline{{\mathscr {F}}(M)}\subseteq {\mathscr {E}}(M)^c$.

First let $x\in {\mathscr {F}}(M)$. Then $x\in {\mathscr {E}}(M)^c$ by definition of ${\mathscr {E}}(M)$. Let $x\in {\mathscr {S}}(M^c)$ and assume $x\notin {\mathscr {E}}(M)^c$, i.e., $x\in {\mathscr {E}}(M)$. Therefore, *x* has a unique nearest point $\zeta$ on *M*. By Lemma 3 there exists $a\in (1,\infty )$ such that $\zeta +a (x-\zeta )$ is the center of the ball $B_{a\Vert x-\zeta \Vert }(\zeta +a (x-\zeta ))\subseteq M^c$, which contains the ball $B_{\Vert x-\zeta \Vert }(x)$. Thus, *x* is not the center of a maximal ball in $M^c$, contradicting $x\in {\mathscr {S}}(M^c)$.

We have shown that ${\mathscr {S}}(M^c) \cup {\mathscr {F}}(M)\subseteq {\mathscr {E}}(M)^c$. Since ${\mathscr {E}}(M)^c$ is closed, also $\overline{{\mathscr {S}}(M^c) \cup {\mathscr {F}}(M)}\subseteq {\mathscr {E}}(M)^c$. $\square$

### *Remark 11*

1. The skeleton ${\mathscr {S}}(M^c)$ is not automatically closed: consider as a counterexample $M=\{(x,|x|)\in {{\mathbb {R}}}^2:x\in {{\mathbb {R}}}\}$, where ${\mathscr {S}}(M^c)=\big \{(0,y):y\in (0,\infty )\big \}$.
2. ${\mathscr {F}}(M)$ is not automatically closed: consider as a counterexample Example 1, where $x\notin {\mathscr {F}}(M)$ but the points on the left of *x*, and belonging to the line through *x* and *p*(*x*) lie in ${\mathscr {F}}(M)$.
3. ${\mathscr {F}}(M)=\emptyset$ for closed *M*. Note further that if *M* is countable with no accumulation points, then ${\mathscr {E}}(M)^c$ is the union of the boundaries of the Voronoi cells corresponding to *M* (see [3, Subsection 6.2.1]).

For the main purpose of this manuscript, closedness of the skeleton has an important consequence.

### **Theorem D**

*If* *M* *is a* $C^{1,1}$ *submanifold and* ${\mathscr {S}}(M^c)$ *is closed*, *then* $\vartheta$ *is continuous*.

Note that by virtue of Theorem D and Corollary 2 we get that ${\mathscr {E}}(M)$ is a fiber bundle if *M* is $C^{1,1}$ with closed ${\mathscr {S}}(M^c)$.

### *Proof*

In view of Theorem A we only need to show that $\vartheta$ is upper semi-continuous. For this we follow the second part of the proof of Theorem A, where we construct, under the assumption that $\vartheta$ is not upper semi-continuous in $(\xi ,v)\in \nu _1(M)$, a sequence $(u_k)_{k\in {{\mathbb {N}}}}$ converging to $z=\xi +\vartheta (\xi ,v)v$, such that for every $k\in {{\mathbb {N}}}$ there exist $\zeta _k,\eta _k\in M$ with $\zeta _k\ne \eta _k$ and $\Vert u_k-\zeta _k\Vert =d(u_k,M)=\Vert u_k-\eta _k\Vert$.

But this implies that $u_k\in {\mathscr {S}}(M^c)$ for all $k\in {{\mathbb {N}}}$. Since ${\mathscr {S}}(M^c)$ is closed by assumption, we also have $z\in {\mathscr {S}}(M^c)$. This means that *z* is the center of a maximal ball in $M^c$.

On the other hand, since $\vartheta$ is not upper semi-continuous in $(\xi ,v)$, there exists $\alpha >\vartheta (\xi ,v)$ such that for all $r\in [\vartheta (\xi ,v),\alpha )$ it holds that $\xi +rv\subseteq {\text {unpp}}(M)$ and $\xi$ is the unique nearest point to $\xi +rv$ on *M* (see again the proof of Theorem A).

But this contradicts the earlier finding that $z=\xi +\vartheta (\xi ,v)v$ is the center of a maximal ball in $M^c$. $\square$

## Appendix: Supplementary proofs

*Proof that* $\nu (M)$ *is a* $C^{k-1}$ *submanifold,* Remark 3 Let $(\xi ,w)\in \nu (M)$. By Proposition 3 and the construction in the according proof (which does not use any conclusion of Remark 3), there is a neighborhood *V* of $\xi$ relative to *M* and $C^{k-1}$ functions $t_1,\ldots ,t_m,n_{m+1},\ldots ,n_d:V\rightarrow {{\mathbb {R}}}^d$ such that $t_1(\zeta ),\ldots ,t_{m}(\zeta )$ are a basis of $T_\xi (M)$ for all $\zeta \in V$ and $(V,n_{m},\ldots ,n_{d})$ is an orthonormal moving frame of $\nu (M)$. Further, there exist open sets ${\tilde{V}}, {\tilde{U}}$ and a $C^k$ diffeomorphism $\varPsi :{\tilde{U}}\rightarrow \tilde{V}$ such that $\xi \in {\tilde{V}}$ and for all $y=(y_1,\ldots ,y_d)\in {\tilde{U}}$ it holds $\varPsi (y)\in M \Longleftrightarrow y_{m+1}=\cdots =y_d=0$. Without loss of generality $V=M\cap {\tilde{V}}$. Then, ${\tilde{V}}\times {{\mathbb {R}}}^d$ is a neighborhood of $(\xi ,w)$. We can now define ${\hat{\varPsi }}:\tilde{W}\times {{\mathbb {R}}}^d\rightarrow \tilde{V}\times {{\mathbb {R}}}^d$ by

5 $$\begin{aligned} (y,\alpha _1,\ldots ,\alpha _d)\mapsto \left( \varPsi (y),\sum _{i=1}^m \alpha _i t_{i}\big (\varPsi (P_{{{\mathbb {R}}}^m}(y))\big )+\sum _{i=m+1}^d \alpha _i n_{i}\big (\varPsi (P_{{{\mathbb {R}}}^m}(y))\big )\right) , \end{aligned}$$

with $P_{{{\mathbb {R}}}^m}$ denoting the projection onto the first *m* coordinates. One can see from the representation (5) that ${\hat{\varPsi }}$ is a $C^{k-1}$ mapping, meeting the requirements of Definition 2 (up to the ordering of the components) showing that $\nu (M)$ is a $C^{k-1}$ submanifold of ${{\mathbb {R}}}^d\times {{\mathbb {R}}}^d$ having dimension $m+(d-m)$. $\square$

*Proof of *Theorem 4 We know from item 2 of Remark 2 that *M* can be represented as the graph of a function on $T_\xi (M)$ in the vicinity of $\xi$, i.e., there exist open sets $W\subseteq T_\xi (M)$ and $U\subseteq T_\xi (M)^\perp$ with $0\in W\cap U$ and a $C^2$ function $\varPhi :W\rightarrow U$ such that $\varPhi (0)=0$ and $M\cap (\xi +W+U)=\{\xi +t+\varPhi (t):t\in W\}$. W.l.o.g. we may identify $T_\xi (M)$ with ${{\mathbb {R}}}^m\times \{0\}$ and $T_\xi (M)^\perp$ with $\{0\}\times {{\mathbb {R}}}^{d-m}$ and we may assume $\varPhi (0)=\xi =0$. Since $\varPhi$ parametrizes *M* over the tangent space in $\xi =0$, we have $D \varPhi (0)=0$. By Proposition 3 there exists an orthonormal moving frame $(V,n_{m+1},\ldots ,n_d)$ of $\nu (V)$ with $V\subset M\cap (W+U)$ open, and $0\in V$, and $v=n_{m+1}(0)$. For $k=m+1,\ldots , d$ let $\varPhi _k$ denote the *k*-th component of $\varPhi$, $\varPhi _{k}(y)=\langle \varPhi (y),n_k(0)\rangle$. Note that $\varPhi _k$ is $C^2$ and $D\varPhi _k(0)=0$, so by Taylor’s theorem

$$\begin{aligned} \varPhi _k(y)=y^\top H_k y+r_k(y) \end{aligned}$$

with $\lim _{y\rightarrow 0} \Vert y\Vert ^{-2}r_k(y)=0$, where $H_k$ is the Hessian of $\varPhi _k$ in 0.

Let $\partial _j$ denote differentiation w.r.t. to the *j*-th coordinate, and let $e_1,\ldots ,e_m$ denote the canonical basis vectors of ${{\mathbb {R}}}^m$. For all $y\in W$ with $y+\varPhi (y)\in V$ we have that $\{\partial _j(y+\varPhi (y)):j=1,\ldots ,m\}=\{(e_j+\partial _j \varPhi (y)):j=1,\ldots ,m\}$ forms a basis of the tangent space of *M* in $y+\varPhi (y)$. In particular,

$$\begin{array}{ll} 0=\left\langle \partial _j(y+\varPhi (y)),n_k(y+\varPhi (y))\right\rangle&\quad \forall j=1,\ldots ,m,\\{}&\quad k=m+1,\ldots ,d \\ \Rightarrow 0=\left\langle \partial _i\partial _j\varPhi (0),n_k(0)\right\rangle +\left\langle e_j,Dn_k(0)e_i\right\rangle &\quad \forall i,j=1,\ldots ,m,\\{}&\quad k=m+1,\ldots ,d \end{array}$$

so that

$$\begin{aligned} \left\langle e_j,-Dn_k(0)e_i\right\rangle =\left\langle \partial _i\partial _j\varPhi (0),n_k(0)\right\rangle =\partial _i\partial _j\varPhi _k(0), \end{aligned}$$

and therefore,

$$\begin{aligned} \left\langle e_j,L_{0,n_k}e_i\right\rangle = \left\langle e_j,-P_{T_0(M)}Dn_k(0)e_i\right\rangle =\partial _i\partial _j\varPhi _k(0)=(H_k)_{ij}. \end{aligned}$$

Thus, the Hessian of $\varPhi _k$ is the matrix representation of the shape operator $L_{0,n_k}$ with respect to the basis $(e_1,\ldots ,e_m)$ of $T_0(M)$. So far we have shown that

$$\begin{aligned} \varPhi (y)=\sum _{k=m+1}^d \langle y, L_{0,n_k} y \rangle n_k(0) + r(y) \end{aligned}$$

with $\lim _{y\rightarrow 0}\Vert y\Vert ^{-2}r(y)=0$ ($r=r_{m+1}n_{m+1}(0)+\cdots +r_d n_d(0)$). In particular, the Hessian is self-adjoint and so is $L_{0,n_k}$, for every $k\in \{m+1,\ldots ,d\}$.

Let $\lambda _1\ge \cdots \ge \lambda _m$ be the eigenvalues of $H_{m+1}$ (and thus of $L_{0,n_{m+1}}$). There is no loss of generality in assuming that $(e_1,\ldots ,e_m)$ are the corresponding eigenvectors of $H_{m+1}$ and thus of $L_{0,n_{m+1}}$.

Case 1 $\lambda _1< 0$. Then for all $R>0$ and for all $z\in B_R(Rn_{m+1}(0))$ we have

$$\begin{aligned} \langle z, Rn_{m+1}(0) \rangle&> \frac{\Vert z\Vert ^2}{2}> 0. \end{aligned}$$

Therefore $$B_R(Rn_{m+1}(0))\subseteq \{z\in {{\mathbb {R}}}^d:\langle z,n_{m+1}(0)\rangle >0 \}$$.

For $\zeta =y+\varPhi (y)\in V\subseteq M$, we have that

$$\begin{aligned} \langle \zeta , n_{m+1}(0)\rangle = \langle y, L_{0,n_{m+1}} y + r_k(y) , n_{m+1}(0)\rangle =\sum _{j=1}^m \lambda _j y_j^2 + r_k(y) <0, \end{aligned}$$

for $\Vert y\Vert$ sufficiently small, and thus $y+\varPhi (y)\notin B_R(Rn_{m+1}(0))$ for such *y*.

Case 2 $\lambda _1> 0$. Let $R<\frac{1}{2\lambda _1}$. Then $\partial B_{R}(R n_{m+1}(0))$ is locally the graph of $n_{m+1}(0)^\perp \rightarrow {{\mathbb {R}}}n_{m+1}(0)$, $y+\sum _{k=m+2}^d w_k n_k(0)\mapsto R-\sqrt{R^2 - \Vert y\Vert ^2-\Vert w\Vert ^2}$. We show that the component of the manifold in direction of $n_{m+1}(0)$ lies below that graph, which implies that the manifold has empty intersection with the interior of $B_{R}(R n_{m+1}(0))$ near 0:

$$\begin{aligned} y+\varPhi (y)&=y+\sum _{k=m+2}^d \varPhi _k(y) n_k(0)+\varPhi _{m+1}(y)n_{m+1}(0)\\&=y+ \sum _{k=m+2}^d \langle y,L_{0,n_{k}(0)} y\rangle n_k(0)+\langle y,L_{0,n_{m+1}(0)} y\rangle n_{m+1}(0) +r(y). \end{aligned}$$

It remains to verify that

6 $$\begin{aligned} R-\sqrt{R^2 - \Vert y\Vert ^2-\sum _{k=m+2}^m\langle y,L_{0,n_{k}(0)} y\rangle ^2} \ge \langle y,L_{0,n_{m+1}(0)}y\rangle + {\tilde{r}}(y) \end{aligned}$$

for $\Vert y\Vert$ sufficiently small with equality iff $y=0$, where the remainder ${\tilde{r}}$ satisfies $\lim _{y\rightarrow 0}\Vert y\Vert ^{-2}\tilde{r}(y)=0$. It is easy to see that for $y\in {{\mathbb {R}}}^m$, $\Vert y\Vert$ small enough ,

$$\begin{aligned} R-\sqrt{R^2 - \Vert y\Vert ^2-\sum _{k=m+2}^m\langle y,L_{0,n_{k}(0)} y\rangle ^2}&\ge R-\sqrt{R^2 - \Vert y\Vert ^2}\\&{\mathop {\ge }\limits ^{(*)}} \lambda _1 \Vert y\Vert ^2 \ge \sum _{k=1}^m \lambda _k y_k^2 =\langle y,L_{0,n_{m+1}(0)}y\rangle , \end{aligned}$$

with equality in $(*)$ iff $y=0$, and (6) follows.

Hence there exists $\varepsilon >0$ with $B_R(R n_{m+1})\cap M \cap B_\varepsilon (0)=\emptyset$.

On the other hand, if $R>\frac{1}{2\lambda _1}$, for all $\varepsilon >0$ we have $B_R(R n_{m+1})\cap M \cap B_\varepsilon (0)\ne \emptyset$. This is obtained by a similar calculation, where one has to note that there exists $a>0$ such that for all *y* with $\Vert y\Vert$ sufficiently small but $\Vert y\Vert \ne 0$,

$$\begin{aligned} R-\sqrt{R^2 - \Vert y\Vert ^2-\sum _{k=m+2}^m\langle y,L_{0,n_{k}(0)} y\rangle ^2}&< R-\sqrt{R^2 - \Vert y\Vert ^2}- a \Vert y\Vert ^2. \end{aligned}$$

Case 3 $\lambda _1=0$. Similar to Case 2, one can show that for all $R>0$ there exists $\varepsilon >0$ such that $B_R(R n_{m+1})\cap M \cap B_\varepsilon (0)=\emptyset$. $\square$

*Proof of *Theorem C Fix $x\in {\mathscr {E}}(M)$ and let $(V,n_{m+1},\ldots ,n_d)$ be an orthonormal moving frame of $\nu (V)$ with $p(x)\in V$.

Step 1 We show that

$$\begin{aligned} \Big ({\text {id}}_{T_{p(x)}(M)}+\!\!\sum _{j=m+1}^d\!\!\big \langle x-p(x) , n_j(p(x))\big \rangle P_{T_{p(x)}(M)}(Dn_j)(p(x))\Big )Dp(x)=P_{T_{p(x)}(M)}. \end{aligned}$$

For this consider a $C^1$ curve $\gamma :(-\varepsilon ,\varepsilon )\rightarrow {\mathscr {E}}(M)$ with $p(\gamma (s))\in V$ for all $s\in (-\varepsilon ,\varepsilon )$ and $\gamma (0)=x$. We can write

$$\begin{aligned} \gamma =p\circ \gamma +(\gamma -p\circ \gamma ) =p\circ \gamma +\!\!\sum _{j}\!\!\big \langle \gamma -p\circ \gamma ,(n_j\circ p\circ \gamma )\big \rangle (n_j\circ p\circ \gamma ), \end{aligned}$$

where for the sake of brevity it is understood that summation ranges from $m+1$ to *d*. Define curves $\beta _j:=n_j\circ p\circ \gamma$ and scalar functions $y_j:=\langle \gamma -p\circ \gamma , \beta _j\rangle$ for $j=m+1,\ldots ,d$, so that $\gamma =p\circ \gamma +\sum _{j}y_j \beta _j$, and abbreviate $P=P_{T_{p(x)}(M)}$. Then

$$\begin{aligned} {\dot{\gamma }}&=(Dp\circ \gamma ) {\dot{\gamma }}+ \sum _j(\dot{y}_j \beta _j+y_j{\dot{\beta }}_j)\\ P{\dot{\gamma }}&=P(Dp\circ \gamma ) {\dot{\gamma }}+ \sum _j (P\dot{y}_j \beta _j + P y_j{\dot{\beta }}_j) =P(Dp\circ \gamma ) {\dot{\gamma }}+ \sum _j (\dot{y}_j P\beta _j + y_j P{\dot{\beta }}_j)\\&=P(Dp\circ \gamma ) {\dot{\gamma }}+ \sum _j(\dot{y}_j P\beta _j + y_j P(Dn_j\circ p\circ \gamma ) (D p\circ \gamma ) {\dot{\gamma }}). \end{aligned}$$

Note that $Dp:T({\mathscr {E}}(M))\rightarrow T(M)$, so *Dp*(*x*) maps into $T_{p(x)}(M)$ and thus $PDp=Dp$. Since $P \beta _j(0)=P n_j\circ p\circ \gamma (0)=0$ and $(Dp\circ \gamma (0)) {\dot{\gamma }}(0)\in T_{p(\gamma (0))}(M)=T_{p(x)}(M)$ and ${\dot{\gamma }}$ can be chosen freely in $T_{p(x)}(M)$, it follows

7 $$\begin{aligned} P&=P(Dp\circ \gamma )(0) + \sum _j y_j(0) P(Dn_j\circ p\circ \gamma )(0)Dp\circ \gamma (0)\nonumber \\&=\Big ({\text {id}}_{T_{p(x)}(M)}+\sum _j\big \langle \gamma (0)-p(\gamma (0)), n_j(p(x))\big \rangle P(Dn_j\circ p\circ \gamma )(0)\Big )Dp\circ \gamma (0)\nonumber \\&=\Big ({\text {id}}_{T_{p(x)}(M)}+\sum _j\big \langle x-p(x), n_j(p(x))\big \rangle P(Dn_j) p(x)\Big )Dp(x). \end{aligned}$$

In particular, in the case where $x\in M$, we see $Dp(x)=P$.

Step 2 We show that

$$\begin{aligned} {\text {id}}_{T_{p(x)}(M)}-\Vert x-p(x)\Vert L_{p(x),v} \end{aligned}$$

is invertible for $\Vert x-p(x)\Vert \ne 0$.

Otherwise there exists $t\in T_{p(x)}(M)$ that is mapped to 0 by ${\text {id}}_{T_{p(x)}(M)}-\Vert x-p(x)\Vert L_{p(x),v}$, and therefore

$$\begin{aligned} t= \Vert x-p(x)\Vert L_{p(x),v}t, \end{aligned}$$

so that *t* is an eigenvector for $L_{p(x), v}$ corresponding to the positive eigenvalue $\Vert x-p(x)\Vert ^{-1}$. It follows from Theorem 4 that $\Vert x-p(x)\Vert \ge \varrho \big (p(x),\tfrac{x-p(x)}{\Vert x-p(x)\Vert }\big )$, which contradicts $x\in {\mathscr {E}}(M)$ by Lemma 5.

Step 3 Conclusion of the proof. For the case where $x\in M$ there is nothing left to show.

In the case where $x\notin M$ we have $\sum _j \langle x-p(x),n_j(p(x))\rangle n_j(p(x))=x-p(x)=\Vert x-p(x)\Vert v$ by the definition of *v*. Furthermore, $v=\sum _j \langle v, n_j(p(x))\rangle n_j(p(x))$, and (*V*, *n*) with $n:=\sum _j \langle v, n_j(p(x))\rangle n_j$ is a unit normal field with $n(p(x))=v$. Thus, $P(Dn)(p(x))=-L_{p(x),v}$ by Definition 7.

On the other hand,

$$\begin{aligned} \Vert x-p(x)\Vert P(Dn)(p(x))&=\Vert x-p(x)\Vert P\Big (D\sum _j\langle v, n_j(p(x))\rangle n_j\Big )(p(x))\\&=\sum _j \Vert x-p(x)\Vert \langle v, n_j(p(x))\rangle P(Dn_j)(p(x))\\&=\sum _j \langle x-p(x), n_j(p(x))\rangle P(Dn_j)(p(x)) \end{aligned}$$

By Steps 2 and 3, we can infer that on $T_{p(x)}(M)$

$$\begin{aligned} Dp(x)&=\Big ({\text {id}}_{T_{p(x)}(M)}-\Vert x-p(x)\Vert L_{p(x),v}\Big )^{-1}, \end{aligned}$$

and since *Dp*(*x*) vanishes on $T_{p(x)}(M)^\perp$,

$$\begin{aligned} Dp(x)&=\Big ({\text {id}}_{T_{p(x)}(M)}-\Vert x-p(x)\Vert L_{p(x),v}\Big )^{-1}P_{T_{p(x)}(M)}\\&=\Big ({\text {id}}_{T_{p(x)}(M)}-\Vert x-p(x)\Vert L_{p(x),v}\Big )^{-1}P_{T_{p(x)}(M)}. \end{aligned}$$

$$\square $$
